# Sarcopenia in chronic kidney disease: what have we learned so far?

**DOI:** 10.1007/s40620-020-00840-y

**Published:** 2020-09-02

**Authors:** Alice Sabatino, Lilian Cuppari, Peter Stenvinkel, Bengt Lindholm, Carla Maria Avesani

**Affiliations:** 1 Division of Nephrology, Department of Medicine and Surgery, University of Parma, Parma, Italy; 2 Division of Nephrology, Federal University of São Paulo and Oswaldo Ramos Foundation, São Paulo, Brazil; 3 Division of Renal Medicine and Baxter Novum, Department of Clinical Science, Technology and Intervention, Karolinska Institute, Stockholm, Sweden; 4 Nutrition Institute, Rio de Janeiro State University, Rio de Janeiro, Brazil

**Keywords:** Sarcopenia, Chronic kidney disease, End stage kidney disease, Skeletal muscle mass, Muscle strength, Physical performance

## Abstract

The term sarcopenia was first introduced in 1988 by Irwin Rosenberg to define a condition of muscle loss that occurs in the elderly. Since then, a broader definition comprising not only loss of muscle mass, but also loss of muscle strength and low physical performance due to ageing or other conditions, was developed and published in consensus papers from geriatric societies. Sarcopenia was proposed to be diagnosed based on operational criteria using two components of muscle abnormalities, low muscle mass and low muscle function. This brought awareness of an important nutritional derangement with adverse outcomes for the overall health. In parallel, many studies in patients with chronic kidney disease (CKD) have shown that sarcopenia is a prevalent condition, mainly among patients with end stage kidney disease (ESKD) on hemodialysis (HD). In CKD, sarcopenia is not necessarily age-related as it occurs as a result of the accelerated protein catabolism from the disease and from the dialysis procedure per se combined with low energy and protein intakes. Observational studies showed that sarcopenia and especially low muscle strength is associated with worse clinical outcomes, including worse quality of life (QoL) and higher hospitalization and mortality rates. This review aims to discuss the differences in conceptual definition of sarcopenia in the elderly and in CKD, as well as to describe etiology of sarcopenia, prevalence, outcome, and interventions that attempted to reverse the loss of muscle mass, strength and mobility in CKD and ESKD patients.

## Introduction

**Fig. 1:**
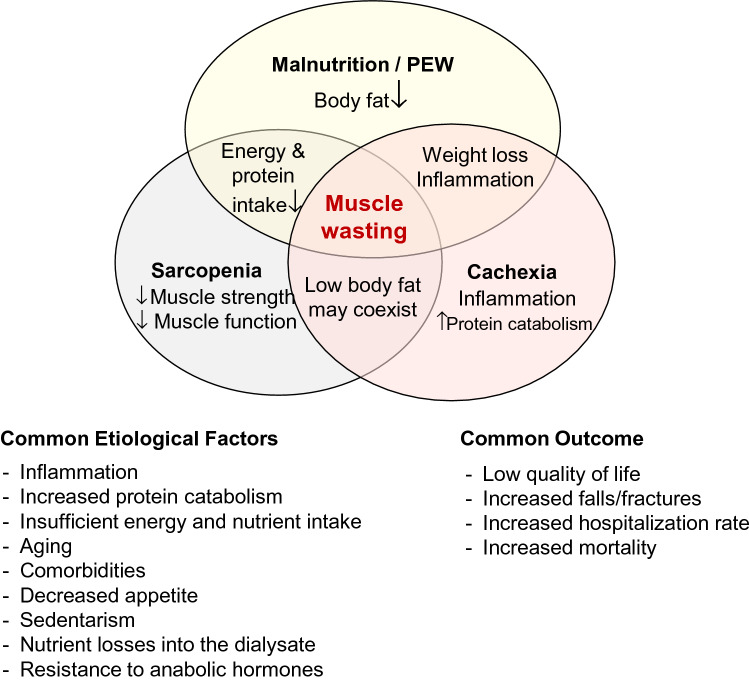
Criteria and clinical outcome of malnutrition/protein energy wasting (PEW), sarcopenia, caquexia and muscle wasting in chronic kidney disease

Loss of muscle mass is a prevalent complication in patients with chronic kidney disease (CKD) and especially in those with end stage kidney disease (ESKD) [1–3]. The causes are diverse and ultimately converge to increased protein degradation and reduced protein synthesis, resulting in a state of negative protein balance [4]. This condition eventually leads to a nutritional disturbance known as protein energy wasting (PEW) that for long has been mostly attributed to malnutrition [5]. In addition to PEW/malnutrition, the terms sarcopenia and cachexia denote nutritional derangements that are related to the loss of muscle mass (wasting) that often is present in CKD. These conditions share common criteria and clinical outcome (Fig. 1) but have distinct definitions. Pure malnutrition is the loss of body weight, muscle mass and body fat due to insufficient energy and nutrient intake, while PEW has similar criteria, but with low-grade inflammation as an additional etiological condition [5]. Sarcopenia, on the other hand, is understood as the concomitant loss of muscle mass and muscle strength that occurs with aging. Cachexia is a syndrome that is present in diseases with chronic inflammation and increased breakdown of muscle proteins, such as in cancer, and is characterized by severe muscle loss that may or may not be accompanied by loss of body fat [6]. These nutritional abnormalities may occur concomitantly depending on the severity of the nutritional impairment. As for example, a patient with malnutrition/PEW may also present sarcopenia, but not necessarily cachexia; while a patient with cachexia may have malnutrition and sarcopenia.

The interest in sarcopenia increased in 2010, with the publication of the sarcopenia consensuses from different societies focusing mainly on the geriatric population [6–10]. Since then, the subject of sarcopenia got the attention of other medical specialties as a condition also present in clinical settings, independent of ageing. Since the publication of the first Sarcopenia Consensus from the European Working Group for Sarcopenia for Older People (EWGSOP) [6], a considerable amount of studies including CKD and ESKD patients on dialysis and kidney transplant recipients has been published. Now is time to analyze the strengths, flaws and applicability of the sarcopenia concept and its relevance for renal care management. This review aims to go through the conceptual definition of sarcopenia, its etiology, prevalence, association with clinical outcomes, and how it is affected by interventions aiming at improving muscle mass, muscle strength and mobility in CKD and ESKD patients.

## Sarcopenia: definition, etiology, operational criteria and methods of assessment

The muscle tissue is one of the main organs in the body. The skeletal muscle is the largest component, but other types, including smooth muscle and cardiac muscle are also part of the muscle compartment. The loss of muscle mass, especially of skeletal muscle mass, is directly associated with diminished strength and indirectly associated with worse quality of life (QoL), increased vulnerability to undesirable outcomes such as falls, loss of independency and, ultimately, higher hospitalization rates and mortality [11]. To the best of our knowledge, Macdonald Critchley in 1931, a neurologist in London, was the first in modern scientific literature to connect the loss of skeletal muscle to ageing when observing that the musculature tends to decrease in the elderly [12]. Since then, many observations were reported regarding the changes in the musculature and body fat that occurs with ageing [13]. These changes refer mainly to an interrelated loss in muscle quantity (mass and volume), decrease in muscle strength and muscle quality, and increase in body fat [14]. Most studies assessing changes in muscularity over life are cross-sectional and the results indicate an estimated decrease in muscle mass of about 1 to 2% per year after the age of 50 years, which tends to further increase after 70 years of age accounting for a total accumulated loss of about 40% between the age of 20 and 70 years [13]. Longitudinal studies including elderly subjects confirm these cross-sectional findings. In septuagenarian individuals, Delmonico et al. [14] demonstrated an annual decrease in muscle area of − 4.9 ± 7.4% in men and of − 3.4 ± 7.9% in women after > 5 years of follow up. A similar result was found by Cameron et al. [15] also in septuagenarian recreationally active men and women, in whom the decrease in thigh lean mass assessed by magnetic resonance imaging (MRI) and whole body lean mass assessed by dual energy x-ray absorptiometry (DXA) was approximately 5% during 5 years. These changes in muscularity have been termed sarcopenia by Irwin Rosenberg in 1988, which comes from Greek and means sarx = flesh and penia = loss [16].

For many years, sarcopenia was mostly understood as loss of muscle mass that occurs with ageing; however, studies showed that not only the muscle quantity, but also muscle strength and physical performance decreases during life [17, 18]. These new findings, conveyed in five sarcopenia consensus papers from different medical societies [6–10], resulted in a common definition in which sarcopenia is a “syndrome characterized by progressive and generalized loss of muscle mass and strength with a risk of adverse outcomes including physical disability, poor QoL and death” [6]. The operational criteria proposed were similar among the consensus reports: sarcopenia is diagnosed when low muscle mass (by measurements of muscle quantity) and low muscle function (by measurements of muscle strength and/or physical performance) occur concomitantly [6–10]. Out of the aforementioned consensuses, the EWGSOP categorized sarcopenia as primary sarcopenia when the etiology is related to aging and as secondary sarcopenia when it results from other conditions that can be concomitant or not with aging and that can occur early in the adult life [6]. Secondary sarcopenia can occur due to low physical activity conditions (bed rest, zero-gravity conditions, sedentary life style), diseases (advanced organ failures disease, inflammatory disease, malignant or endocrine diseases) and nutritional factors (insufficient food intake, malabsorption conditions, gastrointestinal diseases, use of anorexic medications).

The main difference between primary and secondary sarcopenia is that in the first, loss of muscle mass occurs continuously and in a similar fashion after the fourth decade of life, but in the latter, muscle loss is connected not only to ageing but also to conditions that increase protein degradation and therefore is more intense and occurs with greater magnitude than in the natural aging process [19]. In the disease-related secondary sarcopenia, wasting and cachexia are usually present, as is the case in CKD/ESKD, where PEW diagnosed by subjective global assessment or the malnutrition-inflammation score is reported to occur in 11–54% of the patients [20]. Differentiating aging-related from chronic-illness induced sarcopenia is relevant to bring awareness that this phenomenon should be screened in other susceptible groups, such as in young adult CKD patients. In Table 1 we describe the main differences between aging-related and CKD-related sarcopenia; the most important feature that differentiates between the two conditions is the presence of protein degradation in CKD-related sarcopenia, which may be absent in the aging-related sarcopenia. Because of these differences, the treatment goals when treating sarcopenia in elderly individuals may differ from those in individuals with a disease-related condition. In the first group, the main aim is to restore mobility and QoL and not primarily to diminish death rates. In disease-related sarcopenia, where muscle wasting and PEW are more prominent, the main aim of reversing sarcopenia is to recover nutritional status allowing individuals to better respond to the treatment of a determined disease; thus, in addition to reestablishing mobility and QoL, the aim is to diminish the rate of hospitalization and death.

**Table 1:** Comparison between CKD -related sarcopenia and ageing-related sarcopenia in terms of underlying metabolic abnormalities and changes in body fat and muscle fibers

	CKD-related sarcopenia	Ageing-related sarcopenia
Muscle protein degradation	Increased	No change
Muscle protein synthesis	Decreased	Decreased
Resting energy expenditure	Increased/unchanged	Unchanged
Inflammation	Increased	Increased or unchanged
Insulin resistance	Present	Present
Body fat	Unchanged, increased or decreased	Normally increased
Muscle fiber change	Atrophy in type I and II fibers	Preferential loss of type II fibers

After almost 10 years following the publication of the first EWGSOP consensus, a revised consensus was released in 2019 (EWGSOP 2) [10]. Although sarcopenia continued to be assessed by the concomitant presence of low muscle strength and muscle mass, the EWGSOP 2 proposed that low muscle strength should be used as the first measurement to screen for pre-sarcopenia, and low muscle mass and/or poor muscle quality should be used to confirm the sarcopenia diagnosis. If low physical performance is also present, severe sarcopenia is diagnosed.

The shift from low muscle mass to low muscle strength as the key characteristic for the diagnosis of sarcopenia in the EWGSOP 2 is justified by the fact that low muscle strength is better than low muscle mass in predicting worse outcome in the elderly [21]. Moreover, low strength can be easily screened in hospitals, other clinical settings, and community health care, by grip strength using a portable handheld dynamometer. Muscle mass, on the other hand, can be more difficult to evaluate. Many methods enable the assessment of muscle mass, but the method of choice is dependent mainly on the purpose of assessment (research or clinical practice) and one should be aware that differences related to the definition of the tissue assessed can modify the results observed. For instance, fat free mass (FFM), lean body mass/lean soft tissue (LBM/LST), and skeletal muscle mass (SMM) are not equivalent although they are often used as interchangeable surrogates. The FFM, as the name suggests, is the total body mass except the body fat, and it includes the LBM and the bone mineral tissue. The LBM in turn is composed by the total body water, the SMM, and the fat free part of organs [22]. The available methods for the assessment of FFM and its compartments are many, and each one has advantages and disadvantages**.** Different techniques measure different body compartments and identifying the specific body compartment of interest must precede the choice of the method of assessment. Table 2 describes the methods for the assessment of FFM and its components, as well as for muscle strength and physical function.

**Table 2:** Advantages and limitations of methods used for the assessment of fat free mass and its compartments, muscle strength, and physical performance in patients with CKD

Methods	Method	Assessed compartments	Advantages	Limitations
Fat free mass^a^				
Anthropometry	MAMC Calf circumference APMT	SMM	Simple Can be used at the bedside Non-invasive Cheap	Low sensibility Difficulties caused by edema Need for high skilled anthropometrics
Bioelectrical impedance	BIA BIS	FFM TBM ECW	Can be used at the bedside Non-invasive Simple Cheap Provides information regarding nutritional and hydration status	Not a direct measure of lean mass Can be influenced by numerous factors (hydration status, body temperature, nutrition) Available equations that estimate SMM developed in community living elderly people Amputations and pacemakers preclude its use
Physical examination	Visual signs of muscle depletion in specific sites (temple, clavicle, shoulder, scapula/ribs, quadriceps, knee, calf, interosseous)	SMM estimation	Simple Requires no equipment Provides information regarding nutritional status Can be used at bed side	It is not an objective measurement, and therefore, not precise Not sensitive to small changes Requires detailed training
Imaging techniques	CT MRI	SMM	Highly accurate for assessing cross-sectional area and volume CT allows assessment of muscle density which provides information regarding muscle quality Not influenced by hydration status Allows whole body or regional evaluation	Uses ionized radiation (CT) Non-applicable at bedside Very expensive Need for specialized staff Variation between different machines Implanted metal precludes its use (MRI)
	DXA	LBM FFM: LBM + bone ALBM: LBM arms and legs	High precision Total body and regional evaluation Available in many hospitals	Low dose of ionized radiation Non-applicable at bedside Need for specialized staff High fixed cost (low variable costs) Do not differentiate extra-cellular from intra-cellular water
	Ultrasound	SMM	Can be used at the bedside Non-invasive Simple Cheap Reliable and repeatable Echogenicity allows assessment of muscle quality Assessment of cross-sectional area and thickness	Limited data on CKD patients No available cutoffs
Muscle strength				
Grip strength^b^	Handgrip dynamometer	Strength from upper limbs	Simple Portable Cheap Can be easily acquired for use in dialysis clinics, outpatient clinics and hospitals Does not require contribution from the examiner	May not represent strength in the lower limbs May not be suitable for patients with arthritis
Chair stand test	Five chair rise tests	Strength from lower and upper limbs	Simple Does not require equipment Can be easily performed in dialysis clinics, outpatient clinics and hospitals	Requires patient’s collaboration
Physical performance				
Gait speed	Gait speed	NA	Easy to perform Suitable for clinical practice and research Does not requires equipment	Requires a space with a flat corridor
Short physical performance battery (SPPB)	Combination of three scores from tests: balance, gait speed and chair stand^c^	NA	Easy to perform Does not require equipment	Requires the patient’s collaboration Requires a space with a flat corridor
Timed-up-and-go test (TUG)		NA	Easy to perform Does not require equipment	Cutoff points are not extensively validated as compared with gait speed and SPPB Requires a space with a flat corridor
400-m walk or long-distance corridor walk	TUG 400 m walk	NA	Easy to perform Does not require equipment	Requires a space with a flat corridor

*ALBM* Appendicular lean body mass, *APMT* adductor police muscle thicknesses, *BIA* bioelectrical impedance analysis, BIS bioelectrical impedance spectroscopy, *CKD* chronic kidney disease, *CT* computerized tomography, *DXA* dual energy x ray absorptiometry, *ECW* extracellular water, *FFM* fat free mass, *LBM* lean body mass, *MAMC* mid-arm muscle circumference, MRI magnetic resonance imaging, *TBW* total body water, *SMM* skeletal muscle mass, *NA* non-applicable

^a^In order to minimize influence from fluid retention, special care is advised for dialysis patients when assessing fat free mass. For those on hemodialysis, it should be evaluated after the hemodialysis session, and for patients on peritoneal dialysis after the drainage of the dialysis fluid from the peritoneal cavity [166–168]

^b^For patients on hemodialysis, assessment should be preferably performed before the dialysis session when handgrip strength was shown to be higher than after the dialysis session [169]

^c^Each test is scored in 0 to 4 and the maximum score is 12 [170]

## Sarcopenia and CKD

Muscle loss is a frequent finding in CKD, especially for patients with more advanced stages of the disease including ESKD patients undergoing hemodialysis (HD) [1–3]. The consequences of muscle loss are not only related to physical disability as commonly observed in the elderly. In fact, many studies in the past decades have also linked muscle loss in CKD patients with worse QoL, depression, PEW, fracture risk, cardiovascular complications, graft failure and post-operative complications in transplant recipients, as well as with increased hospitalization and mortality [23–30].

**Fig. 2:**
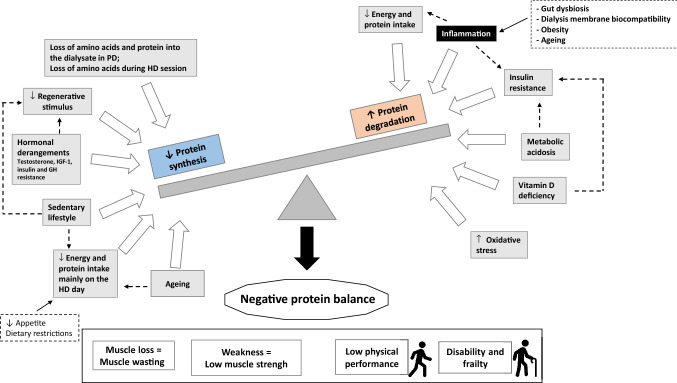
Etiologic factors of muscle derangements leading to muscle loss in chronic kidney disease

The etiologic factors of muscle derangements leading to muscle loss in CKD are diverse and can be related to several conditions including the kidney disease itself, the dialysis procedure and the typical chronic low-grade inflammation present in CKD patients that together increase protein degradation, decrease protein synthesis and lead to a negative protein balance [31, 32] (Fig. 2). The non-inflammatory factors related to the loss of kidney function include the development of metabolic acidosis, insulin resistance and vitamin D deficiency that act as promotors of protein catabolism and decreased protein synthesis [33–38]. Metabolic acidosis acts as a potent stimulator of protein catabolism by triggering two systems responsible for intracellular protein degradation (caspase-3 and the ubiquitin–proteasome systems (UPS)) [39] and by promoting insulin and growth hormone (GH) resistance [40]. Vitamin D deficiency can reduce pancreatic insulin secretion [41, 42], and diminish the stimulus for protein synthesis by decreasing Vitamin D receptors present in muscle and reducing the calcium influx from cellular membranes [38]. Moreover, other factors such as hormonal derangements (testosterone, insulin growth factor (IGF-1) and GH resistance), the substantial loss of amino acids during the HD procedure [43] and reduced energy and protein intake which has shown to be even lower on the dialysis day [44] can also lead to a state of negative energy and protein balance. The inflammatory conditions related to CKD include mainly the pro-inflammatory response induced by the bioincompatibility of the dialysis membranes [4]. More recently, an important role has been attributed to the gastrointestinal tract in the development of inflammation as a consequence of intestinal dysbiosis and barrier disruption [45–47]. This can result from the uremic environment and the reduced fiber intake due to commonly advised dietary restrictions of food sources of potassium, including fruits, vegetables, grains, nuts and whole cereals, which predisposes to an increase in protein fermentation and its metabolites (i.e. ammonium, thiols, phenols, indoles) that accumulates in ESKD patients due to reduced renal clearance [47]. In addition, the gut dysbiosis in uremia may lead to increased exposure to endotoxins that induce inflammatory cascades and systemic low-grade inflammation. Obesity in CKD patients can also act as a pro-inflammatory factor due to adipocyte dysfunction, characterized by increased synthesis of cytokines and chemokines (adipokines) that occurs independently of macrophage infiltration in the adipose tissue, which comes secondarily from adipocyte hypertrophy and hypoxia [48]. Finally, the low physical activity frequently found in HD patients [49] results in “muscle disuse”, which is another important but underappreciated cause of muscle loss and sarcopenia in this population.

Altogether, the conditions that patients with CKD, especially those on dialysis, are exposed to will result in negative protein balance that can result in muscle loss, weakness (low muscle strength), low physical performance, disability and frailty [50] (Fig. 2). Since the CKD population is getting older, ageing is a prominent cause of sarcopenia in ESKD; however, it is likely that this group is more vulnerable to muscle changes than their non-CKD counterparts, but also in comparison to the younger CKD population. This was demonstrated by Çelik et al. [51] who noticed that HD patients aged 65 years and older had lower FFM index, serum creatinine and dry body weight than the younger patients (< 65 years) and by D’Alessandro et al. [52] who showed that sarcopenia was more prevalent in older (> 75 years) than in younger (65–74 years) seniors with CKD stage 3a and 4 (prevalence 55% vs 12.5%, respectively). Of note, the latter study showed that the three components of sarcopenia—skeletal muscle index, handgrip strength (HGS) and performance tests (sit-to-stand-chair-test and 6-min-walk-test) were significantly lower in the older senior CKD patients [52]. A study based on the EQUAL study (*n* = 1334) showed that the risk of PEW in CKD patients with eGFR < 20 ml/min increases substantially with age and is commonly characterized by muscle wasting [53]. Hence, aging adds up as a contributing factor to the etiology of sarcopenia in CKD.

## Prevalence of sarcopenia in CKD: the role of different operational criteria, methods, and cutoffs

For many years, muscle loss was considered part of the malnutrition/PEW syndrome [5]. However, after the publication of the sarcopenia consensuses [6–9], muscle loss due to chronic diseases, such as CKD, became a separate condition to be assessed in the clinical practice. In CKD, the first studies assessing the prevalence of sarcopenia are from 2013 and 2014 [54–58], and only in the last couple of years the scientific literature in this area received many more contributions (Table 3). Up to now, the most used sarcopenia operational criteria was the EWGSOP 1, which was applied in 12 studies [52, 55, 57–66] (sarcopenia prevalence 4–49%), while 4 studies defined sarcopenia only by low muscle mass, not assessing muscle strength or performance [54, 67–69]. An important finding in these studies is that sarcopenia, understood as concomitant low muscle mass and low muscle strength, is a feature of muscle changes in CKD. In addition, another consistent result is that—depending on CKD stage and age, but mostly on the method used to assess muscle mass (DXA, BIA or anthropometry) and the different cutoffs adopted—the prevalence of sarcopenia can be as low as 4% [55, 57] and as high as 63% [70]. These findings suggest that for clinical practice as well as for scientific purposes, there is not yet an agreement on which operational criteria to apply when diagnosing sarcopenia in CKD and dialysis patients.

**Table 3:** Summary of studies on sarcopenia prevalence in non-dialyzed CKD, dialysis, and kidney transplanted patients, and methods and criteria used for establishing presence of sarcopenia

Author	Population	Sample size	Age (years)*	Muscle mass assessment	Strength and/or performance	Sarcopenia Prevalence	Operational criteria used	Observation
CKD Stage 2–5 not on dialysis								
Pereira, NDT 2015 [57]	CKD 3–5	287	59.9 (10.5) 53% > 60 years	MAMC: < 90% reference value SGA—physical exam for low muscle mass BIA: SMMI (< 6.76 kg/m^2^ for women and < 10.76 kg/m^2^ for men)	Strength: HGS (< 30th percentile for sex and age)	MAMC: 9.8% SGA: 9.4% SMMI -BIA: 5.9%	EWGSOP 1	Only SMMI by BIA predicted mortality
De Souza, Plos One 2017 [65]	CKD 2–5	100	73.6 (9.22)	DXA: ASMI (< 7.26 kg/m^2^ for men and < 5.5 kg/m^2^ for women) ASM/BMI (< 0.789 for men and < 0.512 for women)	Strength: HGS (EWGSOP 1: < 30 kg for men and < 20 kg for women; FNIH: < 26 kg for men and < 16 kg for women) Performance: Gait speed (< 0.8 m/s)	FNIH: 28.7% EWGSOP 1: 11.9%	EWGSOP 1 and FNIH	
Ishikawa, Plos One 2018 [171]	CKD 3–5	260	76 (69–80)	DXA: ASMI (< 7.0 kg/m^2^ in males and < 5.4 kg/m^2^ in females)	Strength: HGS (< 26 kg in males and < 18 kg in females) Performance: Gait speed (< 0.8 m/s)	25%	AWGS	Low mass + low strength and/or performance
Hanatani, IJC 2018 [70]	In-hospital CKD	265	72.3 (9.8)	Calf circumference (according to sex and age)	Strength: HGS (according to score chart)	62.6%	Scoring Sarcopenia (sarcopenia score ≥ 105 in men and ≥ 120 in women) by Ishii 2014 [172]	
D’Alessandro, Nutrients 2018 [52]	CKD 3b-4	80 (40 ≥ 75 years, 40 > 60 and < 75 years)	73.7 (7.2) Older: 79.8 (3.3) Younger: 67.5 (4.3)	BIA: SMMI (< 10.75 kg/m^2^)	Strength: HGS (< 30 kg)	Older: 55% Younger: 12%	EWGSOP 1	Only included male patients > 60 years
Vettoretti, Nutrients 2019 [173]	CKD 3b-5	113	80 (6)	Anthropometry: MAMC reduction > 10% of ref population	Strength: HGS (< 26 kg men, < 17 kg women) Performance: Gait speed (severity) (< 0.8 m/s)	24%	EWGSOP 2	Only included patients ≥ 65 years
Fernandes, Nutrition 2019 [64]	CKD 3b-4	73	62.9 (1.1)	DXA: ASMI (< 7.26 kg/m^2^ in men and < 5.5 kg/m^2^ in women)	Strength: HGS (< 30 kg men, < 20 kg women) Performance: Gait speed (< 1 m/s)	12.3%	EWGSOP 1	Sarcopenia defined as low mass + low strength and/or performance
Sharma, CJASN 2014 [54]	CKS 2–4	5192 (CKD 2: 4086, CKD 3a: 775, CKD 3b: 254, CKD 4: 77)	CKD 2: 54.6 (0.4) CKD 3a: 71 (0.6) CKD 3b: 74.5 (1) CKD 4: 73.2 (1.4)	DXA: ASMI (< 7.26 kg/m^2^ in men and < 5.45 kg/m^2^ in women)	NA	CKD 2: < 15% CKD 3a: > 20% CKD 3b: > 20% CKD 4: 34.1%	EWGSOP 1 for low muscle mass	Did not assess strength or performance
Androga, KIRep 2017 [67]	CKD 2–4	1101	70–77	DXA: ASMI (< 7.26 kg/m2 in men and < 5.45 kg/m^2^ in women)	NA	22% (12.5% only sarcopenia and 9.7% obesity sarcopenia)	EWGSOP 1 for low muscle mass	Did not assess strength or performance
Zou, NDT 2017 [66]	CKD 3–5	148	66 (19–87)	NA	Strength: HGS (< 30 kg men, < 20 kg women)	14%	EWGSOP 1	Did not assess muscle mass
Harada, AJC 2017 [68]	CKD	266	71 (62–78)	CT scan: PSOAS area—PSOAS index (< 7.17 cm^2^/m^2^ for man and < 5.13 cm^2^/m^2^ for women)	NA	41.3%	No consensus used to identify sarcopenia	Assessed only muscle mass. Cut-offs defined based on the study area under the ROC curve results
Renal replacement therapy (hemodialysis, peritoneal dialysis and kidney transplantation)								
Kim, CN 2013 [56]	HD	95	63.9 (10)	BIS: LTI (2 standard deviations (SD) or more below the normal gender-specific means for young persons)	Strength: HGS (< 30 kg men, < 20 kg women)	37% in men 29.3% women	EWGSOP 1	
Lamarca, JNHA 2014 [55]	HD	102 (n = 49 for DXA + HGS)	70.7 (7)	DXA: ASMI (< 8.12 kg/m^2^ for men; < 6.08 kg/m^2^ for women; < 6.95 kg/m^2^ for men; < 5.16 kg/m^2^ for women) BIA: LBMI (< 18.1 kg/m^2^ for men; < 14.6 kg/m^2^; < 15.9 kg/m^2^ for men; < 12.8 kg/m^2^ for women) SKF: LBMI (same as LBMI by BIA) MAMC (< 90% standard values) Calf circumference (< 31 cm)	Strength: HGS (< 10th percentile of young individuals according to gender)	From 3.9 to 63.3% depending on the method used to assess muscle mass	EWGSOP 1 and International working group in sarcopenia	Cutoffs: ALMI and LBMI: < 20th percentile of young individuals; < 2 SD below means of young individuals
Isoyama, CJASN 2014 [58]	HD	330	53 (13)	DXA ASMI (< 7.3 kg/m^2^ in men and < 5.5 kg/m^2^ in women)	Strength: HGS (< 30 kg men, < 20 kg women)	20%	EWGSOP 1	ASMI cutoff: two SDs below the sex-specific mean from a young reference population
Kittiskulnam, JCSM 2016 [174]	HD	645	56.7 (14.5)	BIS: SMM and SMMI and SMM/BMI (2SD or more below sex specific means of healthy young adults (18–49 years)	Strength: HGS (< 26 kg for men and < 16 kg for women) Performance: gait speed (< 0.8 m/s)	Using SMMI: 3.9% Using SMM/BMI: 14%	FNIH (for muscle strength and performance)	Reference populations and cutoff points of BIS-derived whole-body muscle mass were obtained from the National Health and Nutrition Examination Survey (NHANES) 2003–2004
Slee, JRN 2019 [59]	HD	87	65.9 (13)	BIA: SMI (< 10.76 kg/m^2^ for men and < 6.76 kg/m^2^ for women) ASMI (< 7.26 kg/m^2^ for men and < 5.45 kg/m^2^ for women) Anthropometry: MAMC (< 23.8 cm for men and < 18.4 cm for women)	Strength: HGS (< 30 kg for men and < 20 kg for women; < 26 kg for men and < 16 kg for women; categorized by BMI and sex based on the Fried criteria [175])	Females EWGSOP: SMMI – 13% ASMI – 17% FNIH: 8% Males EWGSOP: SMMI – 56% ASMI – 30% FNIH: SMMI – 40% ASMI – 22%	EWGSOP 1 and FNIH	Janssen equation for SMM and Sergi equation for ASM calculation
Giglio, JRN 2019 [30]	HD	170	70.6 (7.2)	Baumgartner equation for ASM (previously validated in 47 patients), ASMI calculated (< 7.26 kg/m^2^ for men and < 5.45 kg/m^2^ for women)	Strength: HGS (< 30 kg for men and < 20 kg for women)	36.5%	EWGSOP 1	Sarcopenia defined as low mass + low strength
Kamijo, PDI 2018 [176]	PD	119	66.8 (13.2)	BIS SMI (< 7 kg/m^2^ in men, < 5.7 kg/m^2^ in women)	Strength: HGS (< 26 men, < 18 women) Performance: gait speed (< 0.8 m/s)	11%	AWGS	For diagnosis strength or performance were considered together with muscle mass
Abro, EJCN 2018 [177]	PD	155	63 (14.9)	BIA: ASM derived from segmental BIA (FNIH: < 19.75 kg in men, < 15.02 kg in women) ASM/BMI (FNIH: < 0.789 men, < 0.512 women) ASMI (EWGSOP: < 7.23 kg/m^2^ in men, < 5.67 kg/m^2^ in women; AWGS: < 7 kg/m^2^ in men, < 5.7 kg/m^2^ in women)	Strength: HGS (FNIH: < 26 kg men, < 16 kg women; EWGSOP: < 30 kg men, < 20 kg women; AWGS: < 26 kg men, < 18 kg women)	11–15.5% depending on the criteria	FNIH EWGSOP AWGS	
Hung, NCP 2017 [69]	PD	325	56.7 (16.5)	DXA: ASM (< 2 SD from young population; < 19.75 kg in men and < 15 kg in women) ASMI (< 7.26/ < 7.25/ < 8.5/ < 6.95 kg/m^2^ in men and < 5.75/ < 5.67/ < 5.45/ < 5.16 kg/m^2^ in women ASM/BMI (< 0.789 men; < 0.512 women)	NA	2.2–31.3% for women and 25.1–75.6% for men depending on cutoff used	EWGSOP 1	Only muscle mass evaluation
Da Silva, EJCN 2019 [178]	PD	50	55.7 (16.2)	ASMI (EWGSOP: < 7.26 kg/m^2^ in men, < 5.5 kg/m^2^ in women; EWGSOP 2: < 7 kg/m^2^ in men and < 5.5 kg/m^2^ in women)	Strenght: HGS (EWGSOP: < 30 kg in men, < 20 kg in women; EWGSOP 2: < 27 kg in men, < 16 kg in women) Performance: Gait speed (< 0.8 m/s)	EWGSOP 1: 4% (ASMI + HGS); 4% (ASMI + GS) EWGSOP 2: 4%	EWGSOP 1 and EWGSOP 2	
Yanishi, TP 2016 [179]	KTR	51	46.2 (12.8)	DXA: ASMI (< 7 kg/m^2^ in men, < 5.4 kg/m^2^ in women)	Strenght: HGS (< 27 kg in men, < 18 kg in women) Performance: Gait speed (< 0.8 m/s)	11.8%	AWGS	
Studies including non-dialysis CKD, dialysis and kidney transplanted patients								
Dierkes, BMC 2018 [63]	CKD 3–5 HD KTR	CKD: 112 HD: 24 KTR: 72	CKD: 66 (51–76) HD: 63 (50–76) KTR: 60 (49–67)	BIA: ASMI (≤ 8.87 kg/m^2^ in men and ≤ 6.42 kg/m^2^ in women)	Strength: HGS (< 30 kg men, < 20 kg women)	35% (CKD: 37%, HD: 42%, KTR: 32%)	EWGSOP 1	ALM calculated using MacDonald equation [180]
Wilkinson, Nephrology 2019 [181]	CKD HD KTR	26 CKD 11 HD 35 KTR	55.7 (14.4) CKD: 58.8 (17.8) HD: 61.6 (7.7) KTR: 51.6 (12.2)	DXA: ASM (< 19.75 kg in men and < 15.02 kg in women) ASMI (< 7.26 kg/m^2^ in men and < 5.45 kg/m^2^ in women) ASM/BMI (< 0.789 in men and < 0.512 in women)	Strength: HGS Performance: SPPB (no information regarding cut-offs)	26–35% depending on the muscle mass assessment method	EWGSOP 1 and FNIH	
Lai, Nutrition 2019 [62]	CKD 3–5 Dialysis (HD + PD) KTR	77 (CKD: 26, Dialysis: 37, KTR: 14)	69.6 (9.85)	BIA: SMMI (≤ 10.75 kg/m^2^ in men and ≤ 6.75 kg/m^2^ in women)	Strength: HGS (< 30 kg in men and < 20 kg in women)	49.4%	EWGSOP 1	SMM calculated using Janssen equation [18]

*ALM* appendicular lean mass, *ASM* appendicular skeletal muscle, *ASMI* appendicular skeletal muscle index, *AWGS* Asian working group on sarcopenia, *BIA* bioelectric impedance analysis, *BIS* bioelectric impedance spectroscopy, *BMI* body mass index, *CKD* chronic kidney disease, *DXA* dual energy X-ray absorptiometry, *EWGSOP* European working group on sarcopenia in older people, *FNIH* Foundation for the National Institute of Health, *HD* hemodialysis, *HGS* handgrip strength, *KTR* kidney transplant recipients, *MAMC* mid-arm muscle circumference, *SMMI* skeletal muscle mass index, *SMM* skeletal muscle mass.

*Values in parenthesis describes standard deviation

Also of relevance, is to contextualize the operational definitions of sarcopenia in the general population. These definitions were first developed to predict the risk of mobility loss as well as declines in functional status in the geriatric population. However, accounting for the fact that 92% of older adults have at least one chronic disease [71], the associated effect of ageing and chronic comorbidity must be taken into account when defining sarcopenia. CKD has often been called a model of “accelerated ageing” [72, 73]; therefore, it is likely that the direction of associations between the components of sarcopenia (low muscle mass, strength and performance) and outcomes related to disability and mortality is the same as in the general population. Nevertheless, the magnitude of these relationships is probably different and more pronounced in CKD because of the independent effects of the disease on muscle. In fact, in HD patients, muscle loss occurs at younger ages, and are more marked in comparison to age-matched controls [74, 75]. Therefore, currently used cutoffs to clinically identify sarcopenia in the general population might not be appropriate for CKD including dialysis patients. The definition of what constitutes a low nutritional marker of body composition is dependent on the normal distribution of a nutritional marker in a representative population. For example, in order to define the threshold for low SMM in CKD/ESKD patients, first it would be necessary to search for the distribution of SMM in a representative population of dialysis individuals to investigate the distribution of percentiles of SMM according to age and gender, before finally tracing those with threshold below, for example, the 10th or 5th percentile. In addition, it would also be important to investigate whether SMM at these thresholds is associated with worse outcome. Such a study is difficult to perform and, to the best of our knowledge, has not yet resulted in published work. Studies in CKD/ESKD patients to define appropriate cutoffs to screen for low muscle mass, strength and mobility are scarce. By using computed tomography (CT) to assess muscle mass at the level of the 3^rd^ lumbar vertebra in 233 patients on CKD stages 3–5, Giglio et al. demonstrated that among many surrogate methods tested to assess muscle mass (BIA, MAMC, anthropometry, Janssen and Baumgartner equations, and physical examination of muscle mass from subjective global assessment, SGA), the Baumgartner and Janssen equations (cutoffs for low muscle mass for Baumgartner equation was below 21.4 kg for men and below 14.8 kg for women and for Janssen was 29.3 kg for men and 18.2 kg for women) showed the best agreement in terms of sensitivity, specificity and area under the curve with muscle mass assessed by CT, using the value below the 25th percentile to define low muscle mass (for men 139.1 cm^2^ and for women 97.5 cm^2^) [30]. In a subsequent study with the same cohort, the use of muscle mass assessed by CT at the 3rd lumbar vertebra with the cutoff value below the 25th percentile to define low muscle mass (< 138 cm^2^ for men and < 98 cm^2^ for women) was associated with higher all-cause mortality [76]. For low muscle strength assessed by handgrip strength, when examining 265 dialysis patients (218 HD and 17 peritoneal dialysis (PD) patients) followed for 13.4 ± 7.9 months to analyze mortality, it was shown that the cutoff of handgrip strength best able to predict mortality rate was 22.5 kg for men (61% sensitivity and 76% specificity) and 7 kg for women (83% sensitivity and 37% specificity) [77]. However, since these cutoffs showed a moderate sensitivity for men and a low specificity for women and that ideally, a cutoff to evaluate sarcopenia should screen low values when it can still be reversed and not necessarily when it associates with higher mortality rates, appropriate cutoffs for CKD/ESKD patients are yet to be determined. Until we know better about cutoffs directed to CKD/ESKD, it may be prudent to assume that those proposed in the sarcopenia consensus can be used in CKD patients. However, it is important to acknowledge that cutoffs should be understood as a starting point to screen for patients at risk or who need special medical nutritional attention; thus, continuous follow up using the patient as his/her own control is the best approach to better recognize when an intervention should be implemented as well as to monitor the possible impact in ameliorating poor clinical outcomes.

## Muscle mass, muscle strength or physical function: which one is more clinically relevant for CKD?

Although the three main components of sarcopenia—low muscle mass, low muscle strength and low physical performance—are closely related, they do not necessarily have a causal relation. When sarcopenia was first conceptualized by Rosenberg in 1988, the statement was that the reduction in muscle mass affects ambulation, mobility and weakness, suggesting that loss of muscle mass would lead to low muscle strength and mobility and that the opposite would also occur [16]. However, longitudinal studies following older individuals for 3–5 years showed that the loss of muscle strength occurs more rapidly than the loss of muscle mass [14, 17]. Delmonico et al. [14] showed that among 1880 older individuals (aged 70–79 years), the annual decline in leg muscle strength (~ 3%/year) was 3–5 times higher than the rate of loss in the leg lean mass (~ 1%/year); this pattern of different decline rates occurred in the group that lost as well as in the group that maintained/gained body weight. Of particular interest is the finding that the group that gained body weight had a small increase in lean mass, although muscle strength decreased [14]. These findings suggest that loss of muscle mass is not the only etiologic factor in the loss of muscle strength and thus that other factors are involved. Manini and Clark [78] suggested that conditions related to impairments in neural (central) activation combined with reductions in force-generating capacity of skeletal muscle are involved in the loss of muscle strength [78]. Examples of impaired neural (central) activation are decreased excitatory voluntary stimulus from supraspinal centers and lower or suboptimal motor unit recruitment that cause lower muscle strength. An example of damaged force-generated muscle capacity is that there are changes in actomyosin structure and function with infiltration of adipocytes into muscle fibers that can decrease muscle strength.

Since CKD patients manifest a phenotype of accelerated aging [79], one can expect that similar to changes observed in elderly, a decrease in muscle strength and function are associated, but not only as a result of muscle mass loss. In fact, in a study using an animal model of progressive CKD, it was shown that compared to controls, muscle function of CKD rats decreased although muscle mass did not change. Instead, changes in muscle quality and increased muscle fiber atrophy was observed [80]. In dialysis patients, Fahal et al. [81] investigated changes in muscle weakness by examining quadriceps muscle force and contractile properties, in addition to muscle biopsy with quantification of the type of muscle fibers in HD and PD patients and in healthy controls. The first finding is that muscle strength in dialyzed patients was lower than in healthy controls, and dialyzed patients with malnutrition (assessed by SGA) were weaker than well-nourished patients. Secondly, the most prominent difference between dialysis and controls was the slower relaxation in the muscle, which can compromise muscle strength and contraction, regardless of muscle mass. Third, 78% of the dialysis patients presented some morphologic abnormality in the muscle biopsies, with atrophy in fiber type I (slow-twitch) present in 45% of the patients and atrophy in fiber type II (fast-twitch) in 40% of the patients. Moreover, fiber type II (fast-twitch) area was significantly smaller in the malnourished dialysis group as compared to the well-nourished. These findings corroborate that muscle mass is not the only determinant of muscle strength, but other factors such muscle relaxation and fiber muscle atrophy can also explain a low muscle strength. In fact, in a subsequent cross-sectional study it was demonstrated that compared to age-and sex-matched healthy individuals, HD patients had lower strength, contractive muscle area and gait speed, although the total muscle sectional area was similar to that of healthy matched individuals [82]. These findings support the hypothesis that similar to the elderly, muscle function worsens independently of the progressive loss of muscle mass. A decrease in muscle quality caused by muscle fat infiltration seems to be a missing link. Although studies in this area are scarce, some studies in CKD and HD patients showed that increased muscle fat infiltration in the thigh was associated with lower muscle function assessed by muscle strength and physical performance tests [83, 84]. However, longitudinal studies in CKD/ESKD are warranted to further investigate the role of muscle fat infiltration mediating loss of muscle function.

In addition, in longitudinal studies of patients with ESKD, low muscle strength was a stronger predictor of increased hospitalization and mortality rates than lower muscle mass, reinforcing the idea that low muscle strength is a more powerful determinant of worse outcome [3, 58, 85]. This does not mean, however, that muscle mass should be of less importance when assessing nutritional status, but rather that measurements of muscle strength, that can be easily assessed by HGS, should be incorporated as an important component for the diagnosis of muscle derangements in CKD and ESKD.

## Protein intake: how much is required to avoid muscle wasting in the elderly with CKD?

This question is particularly important for patients with CKD on stages 3 to 5 not on dialysis. The 2020 Updated practice guideline for nutrition in CKD from the Kidney Disease Quality Initiative—National Kidney Foundation (KDOQI-NKF) guidelines recommends a protein intake of 0.6 to 0.8 g/kg/day for patients with CKD on stages 3 to 5 with an energy intake of 30 kcal/kg/day. However, no specific recommendation for elderly subjects with CKD was addressed [86]. For the elderly with CKD, a position paper from the PROT-AGE Study Group, recommends a protein intake of 0.8 g/kg/day for patients with GFR < 30 ml/min and > 0.8 g/kg/day if GFR is between 30 to 60 ml/min [87]. As for elderly not with CKD, a recent guideline recommended a minimum protein intake 1 g /kg/day with 30 kcal/kg/day, but no specific recommendation for elderly with CKD was discussed [88]. These diverse protein recommendations can be explained by the outcome expected. In the elderly without CKD, a higher intake of protein—higher than the intake of 0.8–1.0 g/kg/day recommended for healthy adults—is motivated by findings that protein intake lower than 1 g/kg/day is associated with loss of muscle mass in non-CKD elderly, most likely due to the lower protein synthesis and higher protein degradation rates that are inherent to aging [89]. In individuals with CKD not on dialysis, the recommendation of controlling protein intake aims to reduce the metabolic derangements from the gradual loss of renal function [86]. Therefore, the optimal protein intake for elderly patients with CKD stages 3 to 5 not on dialysis is a controversial subject. The question is—how much protein intake an elderly with CKD not on dialysis should eat to ensure muscle mass preservation and at the same time not further increase the derangements resulting from the loss of renal function? So far, there are no clinical trials addressing this question with conclusive endpoints, such as changes in muscle mass, muscle strength and physical performance. However, interventional studies in patients with CKD (stages 3–5 not on dialysis) evaluating the use of controlled protein intake (moderate to low protein diets—0.6 to 0.8 g/kg/day, or very low protein diet—0.3 to 0.4 g/kg/day supplemented with amino acids or their nitrogen-free keto-analogues) and with adequate energy intake have shown positive findings in elderly CKD patients in preserving good nutritional status [90–93], postponing the beginning of dialysis therapy [90], lowering all-cause mortality [91], good adherence to a moderate restriction in protein intake (0.8 g/kg/day) and increasing serum albumin [92] and better quality of life [93]. More recently, not only the protein amount, but the adherence to higher scores of plant-based diet in elderly men with CKD stages 3–5 was associated with better insulin sensitivity and lower inflammatory markers, supporting the concept that the source and type of protein also plays an important role and has the potential to offer benefits to elderly with CKD [94]. Altogether, these findings are suggestive that controlling the protein intake (0.6–0.8 g/kg/day) in elderly patients with CKD 3–5 can be beneficial but only if an adequate amount of energy is provided as this is needed to prevent impaired protein degradation and the risk of muscle wasting [95]. However, if poor adherence and signs of muscle wasting (indicating malnutrition/PEW, sarcopenia or cachexia) occur, the priority of the dietary scheme should be to interrupt the loss of muscle mass and recover nutritional status. In this case, an energy and protein recommendation of 30 kcal/kg/day and 0.8–1 g/kg/day, respectively, would likely cover for the nutritional needs and can be used as a starting point with subsequent monitoring.

## Can sarcopenia be reversed in CKD?

Classically, nutritional interventions characterized by energy and protein supplementation have been used to improve nutritional intake of malnourished or sarcopenic patients [87, 89]. Evidences suggest that protein supplementation alone may offer limited benefits to older adults in terms of improving muscle mass and strength [96–98] which may be a reflection of the anabolic resistance present in older individuals [99]. On the other hand, studies suggest that the best effect of protein supplementation on muscle protein synthesis occurs when protein supplementation is given immediately after exercise [100, 101]. Despite some data suggesting immediate additional anabolic benefits on combining oral nutritional supplementation and exercise training [102], the available randomized controlled trials (RCT) that investigated long-term effects of both interventions combined, failed to demonstrate any additive benefits on function, strength and muscle mass [103, 104]. Reasons for the lack of positive results may be related to the populations studied being younger than the general dialysis population and had relatively adequate nutritional status; and secondly that the low volume and intensity of the exercise prescribed did not overcome the anabolic resistance that is characteristic of ageing and of HD patients [105–107]. Of note, the exercise load was not aligned with the recommended levels by standard exercise guidelines [108].

Robust evidence in healthy elderly subjects demonstrate the benefits of exercise, particularly resistance training, and physical activity, on muscle mass, strength and performance [109]. The synergistic effects of protein supplementation and exercise to increase protein synthesis and stimulate muscle growth have also been investigated in the elderly. Particularly, resistance exercise in conjunction with increased protein intake may improve the utilization of ingested amino acids for de novo protein synthesis [110, 111]. Similar results were found during prolonged protein supplementation combined with resistance exercise [112]. Results from the renal population are summarized in Table 4.

**Table 4:** Potential effects of exercise training (resistance/endurance) on muscle parameters in CKD/ESKD patients summarized from available evidence

	Resistance training	Aerobic training
Muscle mass	↑↑	–
Muscle strength	↑↑	–
Measures of functional fitness/capacity	↑	↑↑
Performance	↑	↑
Health-related quality of life	–	↑

In the ESKD population, a number of studies have investigated various modalities of exercise in HD patients [113–115]. In comparison to aerobic exercise, that predominantly improves cardio-respiratory endurance and fitness, resistance training promotes muscle growth and strength, and theoretically this may be considered to be the preferred type of exercise to promote physical function in this patient population. Furthermore, the timing of exercise is also of importance in the clinical setting. There are reports suggesting that resistance training during the dialysis session helps to improve compliance to prescribed exercise and could have a positive effect in counteracting the increased catabolism caused by the HD session. An early study from Kopple et al. [106] with 80 HD patients, showed that intradialytic exercise, resistance or endurance, induced transcriptional changes in genes favoring muscle anabolism and improved LBM as assessed by anthropometric parameters [106].

In a single-blind RCT in which 23 HD patients were randomized to progressive resistance exercise training (PRET) or low-intensity lower body stretching activities [116], patients in the intervention group increased thigh muscle volume assessed by MRI, and strength assessed by isometric dynamometer, while patients in the control group presented muscle loss. However, in contrast to findings in the elderly population, no effects on QoL and performance in physical function tests were detected. In a more recent multicenter RCT that tested the effects of a simple personalized walking exercise program at home on functional status of HD patients [107], improvements of the six minutes walking test and the five times sit-to-stand test were described after six months, together with improvements on self-reported QoL.

Regarding the non-dialysis CKD population, fewer studies are available. The RENEXC study compared balance and resistance exercise, both combined with endurance training, in 150 patients with CKD stages 3–5 for four months and reported a significant improvement in muscle strength and physical performance in both groups [117]. In addition, in a pre-specified sub-analysis of the same study with prolonged 12 months duration of the intervention, the effect of both interventions on sarcopenia status was assessed [118]; while no intervention was able to reverse sarcopenia the two groups showed either stabilization (in the resistance exercise group) or improvement (in the balance group) in muscle mass. In the LIFE-P study, elderly subjects were randomized into two groups, physical activity (PA) and a successful aging health education group (SA) for 12–18 months [119]. CKD patients in the PA group had better physical performance results as assessed by the short physical performance battery (SPPB) at 12 months in comparison to patients enrolled in the SA group. While in the ExTra CKD study [120], the addition of resistance exercise to aerobic exercise conferred greater increases in muscle mass and strength in CKD patients than aerobic exercise alone. Finally, in addition to the positive results of exercise in patients on HD reported by Manfredini et al. [107], a recent RCT on exercise in overweight CKD patients by Aoike et al. [121] found that patients who were instructed to perform aerobic exercises at home had similar improvements in functional capacity tests (6 min walking test, 2 min step test, sit to stand, arm curl test, sit-and-reach test, and timed up-and-go), reflecting important improvements in cardiorespiratory fitness, as well as improved QoL and quality of sleep when compared to patients that performed in-center exercises, while no changes in any of the parameters investigated were found in the control group (no exercise).

In summary, available data suggest a possible anabolic resistance in HD patients, probably due to catabolic factors related to the kidney disease per se and disturbances affecting HD patients (uremia, inflammation, acidosis, insulin resistance etc.) [122], which might require a more intensive, comprehensive, and tailored nutrition and exercise prescription to counteract the deleterious consequences of the uremic milieu. Furthermore, presence of these catabolic factors highlights the notion that a one size-fit-all approach may not be equally beneficial for different patients. As discussed above, low muscle mass can be the cause of muscle weakness, which is strongly associated with function and disability; however, muscle mass alone may have no or little direct effect on function and disability [9]. Patients that are weak and have low muscle mass may benefit by interventions that address muscle hypertrophy, such as high load resistance exercise, while weak patients with normal muscle mass may require other strategies. In addition, strength training in CKD was not shown to increase muscle mass, but could improve muscle strength in six out of eight tests and was capable to ameliorate self-rated physical health and physical function assessed by short-form 36 [123]. Future studies should characterize investigated populations in terms of presence of low muscle mass and strength alone or combined, as this distinction may guide the appropriate tailoring of the intervention accordingly.

## Pharmacological interventions

Treatments for sarcopenia have focused mostly on promoting exercise and improving nutritional intake. However, recent scientific advances have brought attention to some potential pharmacological options that will be briefly discussed.

### Amino acids supplementation

CKD and ESKD are characterized by a status of abnormal amino acid (AA) metabolism, particularly involving branched chain amino acids (BCAA) and keto acids (BCKA) [124]. As a consequence, low plasma and cellular levels of BCAA and BCKA are common [124, 125]. BCAAs, particularly leucine, are the most powerful AAs in the stimulation of muscle anabolism and inhibition of catabolism [126]. Several studies have shown that leucine supplementation improves muscle protein synthesis in older adults [127]. Therefore, BCAA supplements were proposed in CKD and ESKD patients to improve muscle synthesis and AA plasma levels. In addition, since protein restriction is a key factor in the conservative management of CKD, essential AA (EAA) and keto-acid (KA) supplements including also BCAA and BCKA were proposed to maintain or improve nutritional status while reducing protein intake as much as possible.

The administration of EAA in malnourished HD patients improved appetite, increased albumin and plasma AA concentrations, and enhanced muscle strength [128–130]. In particular, BCAA supplementation was reported to stimulate appetite and to improve albumin and anthropometric indices [128]. More recently, the use of β-hydroxy-β-methylbutyrate (HMB), a metabolite of leucine that has been shown to attenuate muscle loss in the elderly [131], has been studied in HD patients but there was no apparent benefit of HMB on body composition [132]. Considering the catabolic effects of HD session per se, during which substantial AA losses occur triggering muscle catabolism to maintain constant plasma AA concentration [133, 134], Deleaval et al. [43], performed a pilot cross-over trial in which BCAA enriched dialysis fluids were used to prevent BCAA losses and, consequently, protein catabolism. They found that the intervention increased plasma concentration of valine, isoleucine and leucine, while in the standard dialysate session the mass transfer of amino acids was negative [43]. Regarding patients with CKD not yet on dialysis, nutritional interventions are mainly characterized by a reduction in protein intake in order to minimize uremic toxicity, avoid malnutrition and delay progression of the kidney disease [86]. In this clinical setting, BCKAs are mainly used to fix amine groups and to regenerate BCAAs, with the advantage of being amino-free [135]. The supplementation of very low protein diets (VLPD, 0.3–0.4 g protein/kg/day) and LPD (0.6 g protein/kg/day) with EAA and keto acids is able to maintain a neutral nitrogen balance and body composition [135, 136]. To assess the effect of supplemented VLPDs, Garibotto et al. [136] performed a cross-over trial in which patients had a period of supplemented VLPD and a period of classic LPD. They observed that supplemented VLPD was not associated with changes in body weight and body composition; however, in terms of muscle kinetics, supplemented VLPD was able to reduce the net muscle protein catabolism compared with the classic LPD.

### Blockage of the myostatin and ActRII pathway

Myostatin is a negative regulator of muscle growth via the ActRIIB receptor that is increased in inflamed CKD/ESKD patients [137], and is currently the most investigated target for the development of new drugs intended to block muscle loss and stimulate muscle hypertrophy. The use of a recombinant fusion protein of modified human follistatin (a natural myostatin inhibitor), showed an increase in muscle mass and strength in animal studies [138], but in humans, no effect in muscle strength was observed [139]. The use of anti-myostatin peptibodies led to increased muscle mass and body weight in animal studies [140], as well in humans [141–143]. However, effects on muscle strength were absent or inconsistent, reinforcing the idea that strength is not directly related only to muscle mass, but mainly to the neural system as previously described.

The use of a receptor blockade of both ActRIIA and ActRIIB in humans, resulted in increase in muscle mass and reduced total fat mass [144], and improvement of insulin sensitivity [145]. Effects in muscle strength and function were only described in a proof-of-concept study with improvement of usual gait speed and 6-minute walk distance [146]. In experimental uremia [147], the use of anti-myostatin peptibody for four weeks reversed weight loss and muscle wasting in mice by decreasing protein degradation, increasing protein synthesis and enhancing IGF-signaling and satellite cell function. They also reported a reduction in circulating inflammatory markers. Dong et al. [148], showed that in rodents with CKD, the inhibition of myostatin using a neutralizing peptibody improved muscle fibrosis. Both studies suggest anti-catabolic and anti-inflammatory effects of myostatin inhibitors in experimental uremia.

The results from different trials have shown that blockage of myostatin and ActRII pathways had significant effects on muscle hypertrophy; however, they failed to demonstrate any significant effect on muscle strength and physical function. As discussed above, muscle strength is thought to be the most important parameter in the sarcopenia definition as it is strongly related to disability, hospitalization and mortality. No studies testing these novel drugs on the CKD/ESKD population have been reported.

### Angiotensin II receptor blockers (ARB)

Angiotensin II overexpression is known to intensify muscle catabolism by inhibiting the mTOR pathway, but also to induce protein degradation through the activation of nuclear factor kappa B (NF-κB) and p38 mitogen-activated protein kinase by reactive oxygen species (ROS) accumulation [149]. Cumulative evidence in animal studies reported a protective effect of ARB on skeletal muscle by reducing muscle fibrosis and improving muscle function [150], and a dose dependent enhancement in muscle healing and regeneration [151, 152]. The only available study in humans had a cross-sectional design and reported that ARB use in chronic HD patients was protective, with an independent 75% decrease in the odds of having muscle weakness as assessed by handgrip, when compared to patients who did not use it [153]. Considering these positive results of ARBs that are common drugs patients with CKD/ESKD, further longitudinal and interventional studies are needed to fully clarify the role of ARB in the preservation of muscle mass and strength in ESKD.

### AST-120

AST-120 is an adsorbent used to inhibit the intestinal absorption of indole, *p*-cresol, and food derived advanced glycation end-products [154]. It has been proposed to slow CKD progression as assessed by estimated creatinine clearance in mild and moderate CKD [155] and to improve the uremic syndrome [156]. Uremic toxins, specially indoxyl sulfate have been described as contributors to the chronic inflammation present in ESKD, known to induce skeletal muscle loss [157]. Available evidence in animal and in vitro studies suggest that AST-120 has protective effects on muscle atrophy via the maintenance of mitochondrial function and reduction of the oxidative stress [158]. However, there are no clinical studies showing such effects.

### Ghrelin

Ghrelin, a peptide hormone derived from the gastrointestinal tract that stimulates appetite, increases food intake and promotes fat storage, has been reported to enhance oxygen utilization in skeletal muscle [159]. In nephrectomized mice, the use of acylated ghrelin increased muscle mass and mitochondrial content of muscle [160]. Plasma ghrelin levels are elevated in patients with CKD/ESKD and correlate with fat mass [161]. In a placebo-controlled RCT in malnourished PD patients administration of subcutaneous ghrelin enhanced acute food intake [162]. However, the role if any of ghrelin as a feasible adjunct pharmacologic therapy in patients with PEW/malnutrition, sarcopenia and cachexia remains unclear.

### Ursolic acid

Ursolic acid is a plant compound, found in apple peels, basil leaves, prunes and cranberries. In animal models it has shown beneficial effects in glucose and lipid metabolism [163]. Recently, its effect on skeletal muscle has been investigated in animal models of starvation and denervation [164]. In these models, ursolic acid reversed muscle atrophy by modulating the insulin/IGF-1 signaling. In CKD, a condition with known insulin resistance and IGF-1 deficiency, ursolic acid blocked CKD induced muscle atrophy by suppressing myostatin expression, inflammatory responses associated with NF-κB activations, and by stimulating protein synthesis [165].

## Future perspectives and conclusion

Undoubtedly, sarcopenia is an important nutritional disturbance present in CKD and ESKD that should be routinely screened in clinical practice using one or more of the many available methods (Table 2). CKD-related sarcopenia can occur early in adult life and may develop rapidly as a consequence of the negative energy-protein balance coming from insufficient food intake coupled with increased protein catabolism in patients exposed to the uremic milieu and in HD-patients it may be further enhanced by catabolic effects of the hemodialysis procedure. The prevalence of CKD-related sarcopenia is higher than that observed in ageing-related sarcopenia. It is notable that the prevalence of sarcopenia is higher in HD patients than in non-dialyzed CKD or PD patients, and in kidney transplant recipients (Table 3). Reliable diagnostic methods using models with specific cut-offs for muscle mass and strength that could be used for operational screening for sarcopenia in CKD are however lacking and ought to be developed and tested for validation. Criteria and methods for the diagnosis of sarcopenia should consider the setting (research or clinical practice), the group assessed (CKD, HD, PD or kidney transplant recipients) and periodicity of assessment. Interventions to reverse sarcopenia usually include the use of oral energy and protein supplementation combined with supervised physical resistance exercise (Table 4). However, RCTs show controversial results in reestablishing muscle mass and strength, and in ameliorating physical performance, mobility and QoL. Different study designs, length and type of intervention and primary outcomes make comparisons between studies difficult, but, in general, positive findings in ameliorating one or more components of sarcopenia (muscle mass, muscle strength or physical performance) are observed, with tendency towards more positive effects of ameliorating muscle strength than interventions designed to increase muscle mass. Pharmaceutical interventions aiming at reversing inflammation and protein catabolism have shown promising results in terms of ameliorating CKD-related sarcopenia in experimental settings but are with some exceptions, such as supplementation with amino acids or their keto acid analogues, not routinely used in clinical practice. Investigations with intervention with CKD-related sarcopenia are scarce and the research field is still in its infancy. Moreover, considering the major negative impact on this complication on morbidity and mortality as well as quality of life in patients with CKD/ESKD, further studies are warranted.

## References

[1] Sabatino A, Regolisti G, Delsante M, Di Motta T, Cantarelli C, Pioli S, Grassi G, Batini V, Gregorini M, Fiaccadori E (2019). Noninvasive evaluation of muscle mass by ultrasonography of quadriceps femoris muscle in end-stage renal disease patients on hemodialysis. Clin Nutr.

[2] Foley RN, Wang C, Ishani A, Collins AJ, Murray AM (2007). Kidney function and sarcopenia in the United States general population: NHANES III. Am J Nephrol.

[3] Giglio J, Kamimura M, Lamarca F, Rodrigues J, Santin F, Avesani C (2018). Association of sarcopenia with nutritional parameters, quality of life, hospitalization, and mortality rates of elderly patients on hemodialysis. J Ren Nutr.

[4] Stenvinkel P, Carrero J, von Walden F, Ikizler T, Nader G (2016). Muscle wasting in end-stage renal disease promulgates premature death: established emerging and potential novel treatment strategies. Nephrol Dial Transplant.

[5] Fouque D, Kalantar-Zadeh K, Kopple J, Cano N, Chauveau P, Cuppari L, Franch H, Guarnieri G, Ikizler TA, Kaysen G, Lindholm B, Massy Z, Mitch W, Pineda E, Stenvinkel P, Treviño-Becerra A, Trevinho-Becerra A, Wanner C (2008). A proposed nomenclature and diagnostic criteria for protein-energy wasting in acute and chronic kidney disease. Kidney Int.

[6] Cruz-Jentoft AJ, Baeyens JP, Bauer JM, Boirie Y, Cederholm T, Landi F, Martin FC, Michel JP, Rolland Y, Schneider SM, Topinkova E, Vandewoude M, Zamboni M (2010). Sarcopenia: European consensus on definition and diagnosis: Report of the European Working Group on sarcopenia in older people. Age Ageing.

[7] Muscaritoli M, Anker S, Argilés J, Aversa Z, Bauer J, Biolo G, Boirie Y, Bosaeus I, Cederholm T, Costelli P, Fearon K, Laviano A, Maggio M, Rossi Fanelli F, Schneider S, Schols A, Sieber C (2010). Consensus definition of sarcopenia, cachexia and pre-cachexia: joint document elaborated by Special Interest Groups (SIG) "cachexia-anorexia in chronic wasting diseases" and "nutrition in geriatrics". Clin Nutr.

[8] Fielding RA, Vellas B, Evans WJ, Bhasin S, Morley JE, Newman AB, Abellan van Kan G, Andrieu S, Bauer J, Breuille D, Cederholm T, Chandler J, De Meynard C, Donini L, Harris T, Kannt A, Keime-Guibert F, Onder G, Papanicolaou D, Rolland Y, Rooks D, Sieber C, Souhami E, Verlaan S, Zamboni M (2011). Sarcopenia: an undiagnosed condition in older adults Current consensus definition: prevalence, etiology, and consequences. International working group on sarcopenia. J Am Med Dir Assoc.

[9] Studenski S, Peters K, Alley D, Cawthon P, McLean R, Harris T, Ferrucci L, Guralnik J, Fragala M, Kenny A, Kiel D, Kritchevsky S, Shardell M, Dam T, Vassileva M (2014). The FNIH sarcopenia project: rationale, study description, conference recommendations, and final estimates. J Gerontol A Biol Sci Med Sci.

[10] Cruz-Jentoft AJ, Bahat G, Bauer J, Boirie Y, Bruyere O, Cederholm T, Cooper C, Landi F, Rolland Y, Sayer AA, Schneider SM, Sieber CC, Topinkova E, Vandewoude M, Visser M, Zamboni M (2019). Sarcopenia: revised European consensus on definition and diagnosis. Age Ageing.

[11] Doherty T (2003). Invited review: aging and sarcopenia. J Appl Physiol.

[12] Critchley M (1931). The neurology of old age. Lancet (Lond, Engl).

[13] von Haehling S, Morley J, Anker S (2010). An overview of sarcopenia: facts and numbers on prevalence and clinical impact. J Cachexia Sarcopenia Muscle.

[14] Delmonico M, Harris T, Visser M, Park S, Conroy M, Velasquez-Mieyer P, Boudreau R, Manini T, Nevitt M, Newman A, Goodpaster B (2009). Longitudinal study of muscle strength, quality, and adipose tissue infiltration. Am J Clin Nutr.

[15] Cameron J, McPhee J, Jones D, Degens H (2019). Five-year longitudinal changes in thigh muscle mass of septuagenarian men and women assessed with DXA and MRI. Aging Clin Exp Res.

[16] Rosenberg I (1997). Sarcopenia: origins and clinical relevance. J Nutr.

[17] Goodpaster B, Park S, Harris T, Kritchevsky S, Nevitt M, Schwartz A, Simonsick E, Tylavsky F, Visser M, Newman A (2006). The loss of skeletal muscle strength, mass, and quality in older adults: the health aging and body composition study. J Gerontol A Biol Sci Med Sci.

[18] Janssen I, Baumgartner R, Ross R, Rosenberg I, Roubenoff R (2004). Skeletal muscle cutpoints associated with elevated physical disability risk in older men and women. Am J Epidemiol.

[19] Molfino A, Chiappini MG, Laviano A, Ammann T, Bollea MR, Alegiani F, Rossi Fanelli F, Muscaritoli M (2012). Effect of intensive nutritional counseling and support on clinical outcomes of hemodialysis patients. Nutrition.

[20] Carrero J, Thomas F, Nagy K, Arogundade F, Avesani C, Chan M, Chmielewski M, Cordeiro A, Espinosa-Cuevas A, Fiaccadori E, Guebre-Egziabher F, Hand R, Hung A, Ikizler T, Johansson L, Kalantar-Zadeh K, Karupaiah T, Lindholm B, Marckmann P, Mafra D, Parekh R, Park J, Russo S, Saxena A, Sezer S, Teta D, Ter Wee P, Verseput C, Wang A, Xu H, Lu Y, Molnar M, Kovesdy C (2018). Global prevalence of protein-energy wasting in kidney disease: a meta-analysis of contemporary observational studies from the International Society of Renal Nutrition and Metabolism. J Ren Nutr.

[21] Leong D, Teo K, Rangarajan S, Lopez-Jaramillo P, Avezum A, Orlandini A, Seron P, Ahmed S, Rosengren A, Kelishadi R, Rahman O, Swaminathan S, Iqbal R, Gupta R, Lear S, Oguz A, Yusoff K, Zatonska K, Chifamba J, Igumbor E, Mohan V, Anjana R, Gu H, Li W, Yusuf S (2015). Prognostic value of grip strength: findings from the prospective urban rural epidemiology (PURE) study. Lancet.

[22] Carrero JJ, Johansen KL, Lindholm B, Stenvinkel P, Cuppari L, Avesani CM (2016). Screening for muscle wasting and dysfunction in patients with chronic kidney disease. Kidney Int.

[23] Carrero JJ, Chmielewski M, Axelsson J, Snaedal S, Heimburger O, Barany P, Suliman ME, Lindholm B, Stenvinkel P, Qureshi AR (2008). Muscle atrophy, inflammation and clinical outcome in incident and prevalent dialysis patients. Clin Nutr.

[24] Miyamoto T, Carrero JJ, Qureshi AR, Anderstam B, Heimburger O, Barany P, Lindholm B, Stenvinkel P (2011). Circulating follistatin in patients with chronic kidney disease: implications for muscle strength, bone mineral density, inflammation, and survival. Clin J Am Soc Nephrol.

[25] Martinson M, Ikizler TA, Morrell G, Wei G, Almeida N, Marcus RL, Filipowicz R, Greene TH, Beddhu S (2014). Associations of body size and body composition with functional ability and quality of life in hemodialysis patients. Clin J Am Soc Nephrol.

[26] Beddhu S, Pappas LM, Ramkumar N, Samore M (2003). Effects of body size and body composition on survival in hemodialysis patients. J Am Soc Nephrol.

[27] Noori N, Kopple JD, Kovesdy CP, Feroze U, Sim JJ, Murali SB, Luna A, Gomez M, Luna C, Bross R, Nissenson AR, Kalantar-Zadeh K (2010). Mid-arm muscle circumference and quality of life and survival in maintenance hemodialysis patients. Clin J Am Soc Nephrol.

[28] Streja E, Molnar MZ, Kovesdy CP, Bunnapradist S, Jing J, Nissenson AR, Mucsi I, Danovitch GM, Kalantar-Zadeh K (2011). Associations of pretransplant weight and muscle mass with mortality in renal transplant recipients. Clin J Am Soc Nephrol.

[29] Oterdoom LH, van Ree RM, de Vries AP, Gansevoort RT, Schouten JP, van Son WJ, Homan van der Heide JJ, Navis G, de Jong PE, Gans RO, Bakker SJ (2008). Urinary creatinine excretion reflecting muscle mass is a predictor of mortality and graft loss in renal transplant recipients. Transplantation.

[30] Giglio J, Kamimura M, Souza N, Bichels A, Cordeiro A, Pinho N, Avesani C (2019). Muscle mass assessment by computed tomography in chronic kidney disease patients: agreement with surrogate methods. Eur J Clin Nutr.

[31] Raj DS, Sun Y, Tzamaloukas AH (2008). Hypercatabolism in dialysis patients. Curr Opin Nephrol Hypertens.

[32] Honda H, Qureshi AR, Axelsson J, Heimburger O, Suliman ME, Barany P, Stenvinkel P, Lindholm B (2007). Obese sarcopenia in patients with end-stage renal disease is associated with inflammation and increased mortality. Am J Clin Nutr.

[33] Workeneh BT, Mitch WE (2010). Review of muscle wasting associated with chronic kidney disease. Am J Clin Nutr.

[34] Doucet M, Dube A, Joanisse DR, Debigare R, Michaud A, Pare ME, Vaillancourt R, Frechette E, Maltais F (2010). Atrophy and hypertrophy signalling of the quadriceps and diaphragm in COPD. Thorax.

[35] Testelmans D, Crul T, Maes K, Agten A, Crombach M, Decramer M, Gayan-Ramirez G (2010). Atrophy and hypertrophy signalling in the diaphragm of patients with COPD. Eur Respir J.

[36] Sandri M, Sandri C, Gilbert A, Skurk C, Calabria E, Picard A, Walsh K, Schiaffino S, Lecker SH, Goldberg AL (2004). Foxo transcription factors induce the atrophy-related ubiquitin ligase atrogin-1 and cause skeletal muscle atrophy. Cell.

[37] Crul T, Testelmans D, Spruit MA, Troosters T, Gosselink R, Geeraerts I, Decramer M, Gayan-Ramirez G (2010). Gene expression profiling in vastus lateralis muscle during an acute exacerbation of COPD. Cell Physiol Biochem.

[38] Molina P, Carrero J, Bover J, Chauveau P, Mazzaferro S, Torres P (2017). Vitamin D, a modulator of musculoskeletal health in chronic kidney disease. J Cachexia Sarcopenia Muscle.

[39] Bailey J, Wang X, England B, Price S, Ding X, Mitch W (1996). The acidosis of chronic renal failure activates muscle proteolysis in rats by augmenting transcription of genes encoding proteins of the ATP-dependent ubiquitin-proteasome pathway. J Clin Invest.

[40] Hu Z, Wang H, Lee I, Du J, Mitch W (2009). Endogenous glucocorticoids and impaired insulin signaling are both required to stimulate muscle wasting under pathophysiological conditions in mice. J Clin Invest.

[41] Remuzzi A (2007). Vitamin D, insulin resistance, and renal disease. Kidney Int.

[42] Norman AW, Frankel JB, Heldt AM, Grodsky GM (1980). Vitamin D deficiency inhibits pancreatic secretion of insulin. Science.

[43] Deleaval P, Luaire B, Laffay P, Jambut-Cadon D, Stauss-Grabo M, Canaud B, Chazot C (2020). Short-term effects of branched-chain amino acids-enriched dialysis fluid on branched-chain amino acids plasma level and mass balance: a randomized cross-over study. J Ren Nutr.

[44] Martins A, Dias-Rodrigues J, de Oliveira-Santin F, Barbosa-Brito FDS, Bello-Moreira A, Lourenço R, Avesani C (2015). Food intake assessment of elderly patients on hemodialysis. J Ren Nutr.

[45] Ramezani A, Raj DS (2014). The gut microbiome, kidney disease, and targeted interventions. J Am Soc Nephrol.

[46] Mafra D, Fouque D (2015). Gut microbiota and inflammation in chronic kidney disease patients. Clin Kidney J.

[47] Sabatino A, Regolisti G, Brusasco I, Cabassi A, Morabito S, Fiaccadori E (2015). Alterations of intestinal barrier and microbiota in chronic kidney disease. Nephrol Dial Transplant.

[48] Stenvinkel P, Zoccali C, Ikizler T (2013). Obesity in CKD—what should nephrologists know?. J Am Soc Nephrol.

[49] Regolisti G, Maggiore U, Sabatino A, Gandolfini I, Pioli S, Torino C, Aucella F, Cupisti A, Pistolesi V, Capitanini A, Caloro G, Gregorini M, Battaglia Y, Mandreoli M, Dani L, Mosconi G, Bellizzi V, Di Iorio BR, Conti P, Fiaccadori E (2018). Interaction of healthcare staff's attitude with barriers to physical activity in hemodialysis patients: a quantitative assessment. PLoS ONE.

[50] Cohen S, Nathan JA, Goldberg AL (2015). Muscle wasting in disease: molecular mechanisms and promising therapies. Nat Rev Drug Discov.

[51] Çelik G, Oc B, Kara I, Yılmaz M, Yuceaktas A, Apiliogullari S (2011). Comparison of nutritional parameters among adult and elderly hemodialysis patients. Int J Med Sci.

[52] D'Alessandro C, Piccoli G, Barsotti M, Tassi S, Giannese D, Morganti R, Cupisti A (2018). Prevalence and correlates of sarcopenia among elderly CKD outpatients on tertiary Care. Nutrients.

[53] Windahl K, Faxén Irving G, Almquist T, Lidén M, van de Luijtgaarden M, Chesnaye N, Voskamp P, Stenvinkel P, Klinger M, Szymczak M, Torino C, Postorini M, Drechsler C, Caskey F, Wanner C, Dekker F, Jager K, Evans M (2018). Prevalence and risk of protein-energy wasting assessed by subjective global assessment in older adults with advanced chronic kidney disease: results from the EQUAL study. J Ren Nutr.

[54] Sharma D, Hawkins M, Abramowitz MK (2014). Association of sarcopenia with eGFR and misclassification of obesity in adults with CKD in the United States. Clin J Am Soc Nephrol.

[55] Lamarca F, Carrero J, Rodrigues J, Bigogno F, Fetter R, Avesani C (2014). Prevalence of sarcopenia in elderly maintenance hemodialysis patients: the impact of different diagnostic criteria. J Nutr Health Aging.

[56] Choi JS, Kim YA, Kang YU, Kim CS, Bae EH, Ma SK, Ahn YK, Jeong MH, Kim SW (2013). Clinical impact of hospital-acquired anemia in association with acute kidney injury and chronic kidney disease in patients with acute myocardial infarction. PLoS ONE.

[57] Pereira R, Cordeiro A, Avesani C, Carrero J, Lindholm B, Amparo F, Amodeo C, Cuppari L, Kamimura M (2015). Sarcopenia in chronic kidney disease on conservative therapy: prevalence and association with mortality. Nephrol Dial Transplant.

[58] Isoyama N, Qureshi A, Avesani C, Lindholm B, Bàràny P, Heimbürger O, Cederholm T, Stenvinkel P, Carrero J (2014). Comparative associations of muscle mass and muscle strength with mortality in dialysis patients. Clin J Am Soc Nephrol.

[59] Slee A, McKeaveney C, Adamson G, Davenport A, Farrington K, Fouque D, Kalanter-Zadeh K, Mallett J, Maxwell A, Mullan R, Noble H, O'Donoghue D, Porter S, Seres D, Sheilds J, Witham M, Reid J (2019). Estimating the prevalence of muscle wasting, weakness, and sarcopenia in hemodialysis patients. J Ren Nutr.

[60] Gould D, Watson E, Wilkinson T, Wormleighton J, Xenophontos S, Viana J, Smith A (2019). Ultrasound assessment of muscle mass in response to exercise training in chronic kidney disease: a comparison with MRI. J Cachexia Sarcopenia Muscle.

[61] Menna Barreto A, Barreto Silva M, Pontes K, Costa M, Rosina K, Souza E, Bregman R, Klein M (2019). Sarcopenia and its components in adult renal transplant recipients: prevalence and association with body adiposity. Br J Nutr.

[62] Lai S, Muscaritoli M, Andreozzi P, Sgreccia A, De Leo S, Mazzaferro S, Mitterhofer A, Pasquali M, Protopapa P, Spagnoli A, Amabile M, Molfino A (2019). Sarcopenia and cardiovascular risk indices in patients with chronic kidney disease on conservative and replacement therapy. Nutrition.

[63] Dierkes J, Dahl H, Lervaag Welland N, Sandnes K, Saele K, Sekse I, Marti HP (2018). High rates of central obesity and sarcopenia in CKD irrespective of renal replacement therapy—an observational cross-sectional study. BMC Nephrol.

[64] Fernandes J, Barreto Silva M, Loivos C, Menna Barreto A, Meira V, Kaiser S, Bregman R, Klein M (2019). Obstructive sleep apnea in non-dialyzed chronic kidney disease patients: association with body adiposity and sarcopenia. Nutrition.

[65] Souza V, Oliveira D, Barbosa S, Corrêa J, Colugnati F, Mansur H, Fernandes N, Bastos M (2017). Sarcopenia in patients with chronic kidney disease not yet on dialysis: analysis of the prevalence and associated factors. PLoS ONE.

[66] Zhou Y, Hellberg M, Svensson P, Höglund P, Clyne N (2018). Sarcopenia and relationships between muscle mass, measured glomerular filtration rate and physical function in patients with chronic kidney disease stages 3–5. Nephrol Dial Transplant.

[67] Androga L, Sharma D, Amodu A, Abramowitz M (2017). Sarcopenia, obesity, and mortality in US adults with and without chronic kidney disease. Kidney Int Rep.

[68] Harada K, Suzuki S, Ishii H, Aoki T, Hirayama K, Shibata Y, Negishi Y, Sumi T, Kawashima K, Kunimura A, Shimbo Y, Tatami Y, Kawamiya T, Yamamoto D, Morimoto R, Yasuda Y, Murohara T (2017). Impact of skeletal muscle mass on long-term adverse cardiovascular outcomes in patients with chronic kidney disease. Am J Cardiol.

[69] Hung R, Wong B, Goldet G, Davenport A (2017). Differences in prevalence of muscle wasting in patients receiving peritoneal dialysis per dual-energy X-ray absorptiometry due to variation in guideline definitions of sarcopenia. Nutr Clin Pract.

[70] Hanatani S, Izumiya Y, Onoue Y, Tanaka T, Yamamoto M, Ishida T, Yamamura S, Kimura Y, Araki S, Arima Y, Nakamura T, Fujisue K, Takashio S, Sueta D, Sakamoto K, Yamamoto E, Kojima S, Kaikita K, Tsujita K (2018). Non-invasive testing for sarcopenia predicts future cardiovascular events in patients with chronic kidney disease. Int J Cardiol.

[71] Hung W, Ross J, Boockvar K, Siu A (2011). Recent trends in chronic disease, impairment and disability among older adults in the United States. BMC Geriatr.

[72] Stenvinkel P, Larsson T (2013). Chronic kidney disease: a clinical model of premature aging. Am J Kidney Dis.

[73] Kooman J, Kotanko P, Schols A, Shiels P, Stenvinkel P (2014). Chronic kidney disease and premature ageing. Nat Rev Nephrol.

[74] Domanski M, Ciechanowski K (2012). Sarcopenia: a major challenge in elderly patients with end-stage renal disease. J Aging Res.

[75] Ozkayar N, Altun B, Halil M, Kuyumcu ME, Arik G, Yesil Y, Yildirim T, Yilmaz R, Ariogul S, Turgan C (2014). Evaluation of sarcopenia in renal transplant recipients. Nephrourol Mon.

[76] Bichels A, Cordeiro A, Avesani CM, Amparo F, Giglio J, Souza N, Pinho N, Amodeo C, Carrero JJ, Lindholm B, Stenvinkel P, Kamimura MA (2020). Muscle mass assessed by computed tomography at the third lumbar vertebra predicts patient survival in chronic kidney disease. J Ren Nutr.

[77] Vogt B, Borges M, Goés C, Caramori J (2016). Handgrip strength is an independent predictor of all-cause mortality in maintenance dialysis patients. Clin Nutr.

[78] Clark B, Manini T (2012). What is dynapenia?. Nutrition.

[79] Gracia-Iguacel C, Qureshi A, Avesani C, Heimbürger O, Huang X, Lindholm B, Bárány P, Ortiz A, Stenvinkel P, Carrero J (2013). Subclinical versus overt obesity in dialysis patients: more than meets the eye. Nephrol Dial Transplant.

[80] Organ J, Srisuwananukorn A, Price P, Joll J, Biro K, Rupert J, Chen N, Avin K, Moe S, Allen M (2016). Reduced skeletal muscle function is associated with decreased fiber cross-sectional area in the Cy/+ rat model of progressive kidney disease. Nephrol Dial Transplant.

[81] Fahal I, Bell G, Bone J, Edwards R (1997). Physiological abnormalities of skeletal muscle in dialysis patients. Nephrol Dial Transplant.

[82] Johansen K, Shubert T, Doyle J, Soher B, Sakkas G, Kent-Braun J (2003). Muscle atrophy in patients receiving hemodialysis: effects on muscle strength, muscle quality, and physical function. Kidney Int.

[83] Cheema B, Abas H, Smith B, O'Sullivan A, Chan M, Patwardhan A, Kelly J, Gillin A, Pang G, Lloyd B, Berger K, Baune B, Singh M (2010). Investigation of skeletal muscle quantity and quality in end-stage renal disease. Nephrology.

[84] Wilkinson T, Gould D, Nixon D, Watson E, Smith A (2019). Quality over quantity? Association of skeletal muscle myosteatosis and myofibrosis on physical function in chronic kidney disease. Nephrol Dial Transplant.

[85] Kittiskulnam P, Chertow G, Carrero J, Delgado C, Kaysen G, Johansen K (2017). Sarcopenia and its individual criteria are associated, in part with mortality among patients on hemodialysis. Kidney Int.

[86] Ikizler TA, Burrowes JD, Byham-Gray LD, Campbell KL, Carrero JJ, Chan W, Fouque D, Friedman AN, Ghaddar S, Goldstein-Fuchs DJ, Kaysen GA, Kopple JD, Teta D, Yee-Moon Wang A, Cuppari L (2020). KDOQI nutrition in CKD guideline work group. KDOQI clinical practice guideline for nutrition in CKD: 2020 update. Am J Kidney Dis.

[87] Bauer J, Biolo G, Cederholm T, Cesari M, Cruz-Jentoft A, Morley J, Phillips S, Sieber C, Stehle P, Teta D, Visvanathan R, Volpi E, Boirie Y (2013). Evidence-based recommendations for optimal dietary protein intake in older people: a position paper from the PROT-AGE study group. J Am Med Dir Assoc.

[88] Volkert D, Beck A, Cederholm T, Cruz-Jentoft A, Goisser S, Hooper L, Kiesswetter E, Maggio M, Raynaud-Simon A, Sieber C, Sobotka L, van Asselt D, Wirth R, Bischoff S (2019). ESPEN guideline on clinical nutrition and hydration in geriatrics. Clin Nutr.

[89] Deutz N, Bauer J, Barazzoni R, Biolo G, Boirie Y, Bosy-Westphal A, Cederholm T, Cruz-Jentoft A, Krznariç Z, Nair K, Singer P, Teta D, Tipton K, Calder P (2014). Protein intake and exercise for optimal muscle function with aging: recommendations from the ESPEN expert group. Clin Nutr.

[90] Brunori G, Viola B, Parrinello G, De Biase V, Como G, Franco V, Garibotto G, Zubani R, Cancarini G (2007). Efficacy and safety of a very-low-protein diet when postponing dialysis in the elderly: a prospective randomized multicenter controlled study. Am J Kidney Dis.

[91] Watanabe D, Machida S, Matsumoto N, Shibagaki Y, Sakurada T (2018). Age modifies the association of dietary protein intake with all-cause mortality in patients with chronic kidney disease. Nutrients.

[92] Fois A, Chatrenet A, Cataldo E, Lippi F, Kaniassi A, Vigreux J, Froger L, Mongilardi E, Capizzi I, Biolcati M, Versino E, Piccoli G (2018). Moderate protein restriction in advanced CKD: a feasible option in an elderly high-comorbidity population. A stepwise multiple-choice system approach. Nutrients.

[93] Piccoli G, Di Iorio B, Chatrenet A, D'Alessandro C, Nazha M, Capizzi I, Vigotti F, Fois A, Maxia S, Saulnier P, Cabiddu G, Cupisti A (2020). Dietary satisfaction and quality of life in chronic kidney disease patients on low-protein diets: a multicentre study with long-term outcome data (TOrino-Pisa Study). Nephrol Dial Transplant.

[94] González-Ortiz A, Xu H, Avesani C, Lindholm B, Cederholm T, Risérus U, Ärnlöv J, Espinosa-Cuevas A, Carrero J (2020). Plant-based diets, insulin sensitivity and inflammation in elderly men with chronic kidney disease. J Nephrol.

[95] Garibotto G, Picciotto D, Saio M, Esposito P, Verzola D (2020). Muscle protein turnover and low-protein diets in patients with chronic kidney disease. Nephrol Dial Transplant.

[96] Arnarson A, Gudny Geirsdottir O, Ramel A, Briem K, Jonsson P, Thorsdottir I (2013). Effects of whey proteins and carbohydrates on the efficacy of resistance training in elderly people: double blind, randomised controlled trial. Eur J Clin Nutr.

[97] Weisgarber K, Candow D, Farthing J (2015). Whey protein and high-volume resistance training in postmenopausal women. J Nutr Health Aging.

[98] Bhasin S, Apovian C, Travison T, Pencina K, Moore L, Howland A, Chen R, Knapp P, Singer M, Shah M, Secinaro K, Eder R, Hally K, Schram H, Bearup R, Beleva Y, McCarthy A, Woodbury E, McKinnon J, Fleck G, Storer T, Basaria S (2018). Effect of protein intake on lean body mass in functionally limited older men: a randomized clinical trial. JAMA Intern Med.

[99] Smith G, Atherton P, Reeds D, Mohammed B, Rankin D, Rennie M, Mittendorfer B (2011). Dietary omega-3 fatty acid supplementation increases the rate of muscle protein synthesis in older adults: a randomized controlled trial. Am J Clin Nutr.

[100] Witard O, Jackman S, Breen L, Smith K, Selby A, Tipton K (2014). Myofibrillar muscle protein synthesis rates subsequent to a meal in response to increasing doses of whey protein at rest and after resistance exercise. Am J Clin Nutr.

[101] Smiles W, Areta J, Coffey V, Phillips S, Moore D, Stellingwerff T, Burke L, Hawley J, Camera D (2015). Modulation of autophagy signaling with resistance exercise and protein ingestion following short-term energy deficit. Am J Physiol Regul Integr Comp Physiol.

[102] Pupim L, Flakoll P, Levenhagen D, Ikizler T (2004). Exercise augments the acute anabolic effects of intradialytic parenteral nutrition in chronic hemodialysis patients. Am J Physiol Endocrinol Metab.

[103] Martin-Alemañy G, Valdez-Ortiz R, Olvera-Soto G, Gomez-Guerrero I, Aguire-Esquivel G, Cantu-Quintanilla G, Lopez-Alvarenga J, Miranda-Alatriste P, Espinosa-Cuevas A (2016). The effects of resistance exercise and oral nutritional supplementation during hemodialysis on indicators of nutritional status and quality of life. Nephrol Dial Transplant.

[104] Jeong J, Biruete A, Tomayko E, Wu P, Fitschen P, Chung H, Ali M, McAuley E, Fernhall B, Phillips S, Wilund K (2019). Results from the randomized controlled IHOPE trial suggest no effects of oral protein supplementation and exercise training on physical function in hemodialysis patients. Kidney Int.

[105] Koh K, Fassett R, Sharman J, Coombes J, Williams A (2010). Effect of intradialytic versus home-based aerobic exercise training on physical function and vascular parameters in hemodialysis patients: a randomized pilot study. Am J Kidney Dis.

[106] Kopple J, Wang H, Casaburi R, Fournier M, Lewis M, Taylor W, Storer T (2007). Exercise in maintenance hemodialysis patients induces transcriptional changes in genes favoring anabolic muscle. J Am Soc Nephrol.

[107] Manfredini F, Mallamaci F, D'Arrigo G, Baggetta R, Bolignano D, Torino C, Lamberti N, Bertoli S, Ciurlino D, Rocca-Rey L, Barillà A, Battaglia Y, Rapanà R, Zuccalà A, Bonanno G, Fatuzzo P, Rapisarda F, Rastelli S, Fabrizi F, Messa P, De Paola L, Lombardi L, Cupisti A, Fuiano G, Lucisano G, Summaria C, Felisatti M, Pozzato E, Malagoni A, Castellino P, Aucella F, Abd ElHafeez S, Provenzano P, Tripepi G, Catizone L, Zoccali C (2017). Exercise in patients on dialysis: a multicenter randomized clinical trial. J Am Soc Nephrol.

[108] Nelson M, Rejeski W, Blair S, Duncan P, Judge J, King A, Macera C, Castaneda-Sceppa C (2007). Physical activity and public health in older adults: recommendation from the American College of Sports Medicine and the American Heart Association. Med Sci Sports Exerc.

[109] Jadczak A, Makwana N, Luscombe-Marsh N, Visvanathan R, Schultz T (2018). Effectiveness of exercise interventions on physical function in community-dwelling frail older people: an umbrella review of systematic reviews. JBI Database Syst Rev Implement Rep.

[110] Moore D, Tang J, Burd N, Rerecich T, Tarnopolsky M, Phillips S (2009). Differential stimulation of myofibrillar and sarcoplasmic protein synthesis with protein ingestion at rest and after resistance exercise. J Physiol.

[111] Pennings B, Koopman R, Beelen M, Senden J, Saris W, van Loon L (2011). Exercising before protein intake allows for greater use of dietary protein-derived amino acids for de novo muscle protein synthesis in both young and elderly men. Am J Clin Nutr.

[112] Cermak N, Res P, de Groot L, Saris W, van Loon L (2012). Protein supplementation augments the adaptive response of skeletal muscle to resistance-type exercise training: a meta-analysis. Am J Clin Nutr.

[113] Johansen K (2007). Exercise in the end-stage renal disease population. J Am Soc Nephrol.

[114] Salhab N, Karavetian M, Kooman J, Fiaccadori E, El Khoury C (2019). Effects of intradialytic aerobic exercise on hemodialysis patients: a systematic review and meta-analysis. J Nephrol.

[115] Calella P, Hernández-Sánchez S, Garofalo C, Ruiz J, Carrero J, Bellizzi V (2019). Exercise training in kidney transplant recipients: a systematic review. J Nephrol.

[116] Kirkman D, Mullins P, Junglee N, Kumwenda M, Jibani M, Macdonald J (2014). Anabolic exercise in haemodialysis patients: a randomised controlled pilot study. J Cachexia Sarcopenia Muscle.

[117] Hellberg M, Höglund P, Svensson P, Clyne N (2018). Comparing effects of 4 months of two self-administered exercise training programs on physical performance in patients with chronic kidney disease: RENEXC—A randomized controlled trial. PLoS ONE.

[118] Zhou Y, Hellberg M, Hellmark T, Höglund P, Clyne N (2019). Muscle mass and plasma myostatin after exercise training: a substudy of renal exercise (RENEXC)—a randomized controlled trial. Nephrol Dial Transplant.

[119] Liu C, Milton J, Hsu F, Beavers K, Yank V, Church T, Shegog J, Kashaf S, Nayfield S, Newman A, Stafford R, Nicklas B, Weiner D, Fielding R (2017). The effect of chronic kidney disease on a physical activity intervention: impact on physical function, adherence, and safety. J Clin Nephrol Ren Care.

[120] Watson E, Gould D, Wilkinson T, Xenophontos S, Clarke A, Vogt B, Viana J, Smith A (2018). Twelve-week combined resistance and aerobic training confers greater benefits than aerobic training alone in nondialysis CKD. Am J Physiol Renal Physiol.

[121] Aoike D, Baria F, Kamimura M, Ammirati A, Cuppari L (2018). Home-based versus center-based aerobic exercise on cardiopulmonary performance, physical function, quality of life and quality of sleep of overweight patients with chronic kidney disease. Clin Exp Nephrol.

[122] van Vliet S, Skinner S, Beals J, Pagni B, Fang H, Ulanov A, Li Z, Paluska S, Mazzulla M, West D, Moore D, Wilund K, Burd N (2018). Dysregulated handling of dietary protein and muscle protein synthesis after mixed-meal ingestion in maintenance hemodialysis patients. Kidney Int Rep.

[123] Molsted S, Bjørkman A (2019). Lundstrøm L (2019) Effects of strength training to patients undergoing dialysis: a systematic review. Dan Med J..

[124] Cano N, Fouque D, Leverve X (2006). Application of branched-chain amino acids in human pathological states: renal failure. J Nutr.

[125] Małgorzewicz S, Debska-Slizień A, Rutkowski B, Lysiak-Szydłowska W (2008). Serum concentration of amino acids versus nutritional status in hemodialysis patients. J Ren Nutr.

[126] Gibson N, Fereday A, Cox M, Halliday D, Pacy P, Millward D (1996). Influences of dietary energy and protein on leucine kinetics during feeding in healthy adults. Am J Physiol.

[127] Gielen E, Beckwée D, Delaere A, De Breucker S, Vandewoude M, Bautmans I (2020). Nutritional interventions to improve muscle mass, muscle strength, and physical performance in older people: an umbrella review of systematic reviews and meta-analyses. Nutr Rev.

[128] Hiroshige K, Sonta T, Suda T, Kanegae K, Ohtani A (2001). Oral supplementation of branched-chain amino acid improves nutritional status in elderly patients on chronic haemodialysis. Nephrol Dial Transplant.

[129] Acchiardo S, Moore L, Cockrell S (1982). Effect of essential amino acids (EAA) on chronic hemodialysis (CHD) patients (PTS). Trans Am Soc Artif Intern Organs.

[130] Eustace J, Coresh J, Kutchey C, Te P, Gimenez L, Scheel P, Walser M (2000). Randomized double-blind trial of oral essential amino acids for dialysis-associated hypoalbuminemia. Kidney Int.

[131] Wu H, Xia Y, Jiang J, Du H, Guo X, Liu X, Li C, Huang G, Niu K (2015). Effect of beta-hydroxy-beta-methylbutyrate supplementation on muscle loss in older adults: a systematic review and meta-analysis. Arch Gerontol Geriatr.

[132] Fitschen P, Biruete A, Jeong J, Wilund K (2017). Efficacy of beta-hydroxy-beta-methylbutyrate supplementation in maintenance hemodialysis patients. Hemodial Int.

[133] Chazot C, Shahmir E, Matias B, Laidlaw S, Kopple J (1997). Dialytic nutrition: provision of amino acids in dialysate during hemodialysis. Kidney Int.

[134] Ikizler T, Flakoll P, Parker R, Hakim R (1994). Amino acid and albumin losses during hemodialysis. Kidney Int.

[135] Cupisti A, Bolasco P (2017). Keto-analogues and essential aminoacids and other supplements in the conservative management of chronic kidney disease. Panminerva Med.

[136] Garibotto G, Sofia A, Parodi E, Ansaldo F, Bonanni A, Picciotto D, Signori A, Vettore M, Tessari P, Verzola D (2018). Effects of low-protein, and supplemented very low-protein diets, on muscle protein turnover in patients with CKD. Kidney Int Rep.

[137] Yano S, Nagai A, Isomura M, Yamasaki M, Kijima T, Takeda M, Hamano T, Nabika T (2015). Relationship between blood myostatin levels and kidney function: Shimane CoHRE study. PLoS ONE.

[138] Pearsall R, Widrick J, Cotton E, Sako D, Liu J, Davies M, Heveron K, Maguire M, Castonguay R, Krishnan L, Troy M, Liharska K, Steeves R, Strand J, Keefe T, Cannell M, Alimzhanov M, Grinberg A, Kumar R (2015). ACE-083 increases muscle hypertrophy and strength in C57BL/6 mice. Neuromuscul Disord.

[139] Glasser C, Gartner M, Wilson D, Miller B, Sherman M, Attie K (2018). Locally acting ACE-083 increases muscle volume in healthy volunteers. Muscle Nerve.

[140] St-Andre M, Johnson M, Bansal P, Wellen J, Robertson A, Opsahl A, Burch P, Bialek P, Morris C, Owens J (2017). A mouse anti-myostatin antibody increases muscle mass and improves muscle strength and contractility in the mdx mouse model of duchenne muscular dystrophy and its humanized equivalent, domagrozumab (PF-06252616), increases muscle volume in cynomolgus monkeys. Skeletal Muscle.

[141] Becker C, Lord S, Studenski S, Warden S, Fielding R, Recknor C, Hochberg M, Ferrari S, Blain H, Binder E, Rolland Y, Poiraudeau S, Benson C, Myers S, Hu L, Ahmad Q, Pacuch K, Gomez E, Benichou O (2015). Myostatin antibody (LY2495655) in older weak fallers: a proof-of-concept, randomised, phase 2 trial. Lancet Diabetes Endocrinol.

[142] Bhattacharya I, Pawlak S, Marraffino S, Christensen J, Sherlock S, Alvey C, Morris C, Arkin S, Binks M (2018). Safety, tolerability, pharmacokinetics, and pharmacodynamics of domagrozumab (PF-06252616) an antimyostatin monoclonal antibody in healthy subjects. Clin Pharmacol Drug Dev.

[143] Woodhouse L, Gandhi R, Warden S, Poiraudeau S, Myers S, Benson C, Hu L, Ahmad Q, Linnemeier P, Gomez E, Benichou O (2016). A phase 2 randomized study investigating the efficacy and safety of myostatin antibody LY2495655 versus placebo in patients undergoing elective total hip arthroplasty. J Frailty Aging.

[144] Garito T, Zakaria M, Papanicolaou D, Li Y, Pinot P, Petricoul O, Laurent D, Rooks D, Rondon J, Roubenoff R (2018). Effects of bimagrumab, an activin receptor type II inhibitor, on pituitary neurohormonal axes. Clin Endocrinol.

[145] Garito T, Roubenoff R, Hompesch M, Morrow L, Gomez K, Rooks D, Meyers C, Buchsbaum M, Neelakantham S, Swan T, Filosa L, Laurent D, Petricoul O, Zakaria M (2018). Bimagrumab improves body composition and insulin sensitivity in insulin-resistant individuals. Diabetes Obes Metab.

[146] Rooks D, Praestgaard J, Hariry S, Laurent D, Petricoul O, Perry R, Lach-Trifilieff E, Roubenoff R (2017). Treatment of sarcopenia with bimagrumab: results from a phase II randomized controlled proof-of-concept study. J Am Geriatr Soc.

[147] Zhang L, Rajan V, Lin E, Hu Z, Han H, Zhou X, Song Y, Min H, Wang X, Du J, Mitch W (2011). Pharmacological inhibition of myostatin suppresses systemic inflammation and muscle atrophy in mice with chronic kidney disease. FASEB J.

[148] Dong J, Dong Y, Chen Z, Mitch W, Zhang L (2017). The pathway to muscle fibrosis depends on myostatin stimulating the differentiation of fibro/adipogenic progenitor cells in chronic kidney disease. Kidney Int.

[149] Cabello-Verrugio C, Morales M, Rivera J, Cabrera D, Simon F (2015). Renin-angiotensin system: an old player with novel functions in skeletal muscle. Med Res Rev.

[150] Burks T, Andres-Mateos E, Marx R, Mejias R, Van Erp C, Simmers J, Walston J, Ward C, Cohn R (2011). Losartan restores skeletal muscle remodeling and protects against disuse atrophy in sarcopenia. Sci Transl Med.

[151] Yabumoto C, Akazawa H, Yamamoto R, Yano M, Kudo-Sakamoto Y, Sumida T, Kamo T, Yagi H, Shimizu Y, Saga-Kamo A, Naito A, Oka T, Lee J, Suzuki J, Sakata Y, Uejima E, Komuro I (2015). Angiotensin II receptor blockade promotes repair of skeletal muscle through down-regulation of aging-promoting C1q expression. Sci Rep.

[152] Bedair H, Karthikeyan T, Quintero A, Li Y, Huard J (2008). Angiotensin II receptor blockade administered after injury improves muscle regeneration and decreases fibrosis in normal skeletal muscle. Am J Sports Med.

[153] Lin Y, Chen S, Lai Y, Wang C, Kuo C, Liou H, Hsu B (2019). Angiotensin II receptor blockade is associated with preserved muscle strength in chronic hemodialysis patients. BMC Nephrol.

[154] Goto S, Yoshiya K, Kita T, Fujii H, Fukagawa M (2011). Uremic toxins and oral adsorbents. Therap Apheresis Dial.

[155] Nishikawa M, Ishimori N, Takada S, Saito A, Kadoguchi T, Furihata T, Fukushima A, Matsushima S, Yokota T, Kinugawa S, Tsutsui H (2015). AST-120 ameliorates lowered exercise capacity and mitochondrial biogenesis in the skeletal muscle from mice with chronic kidney disease via reducing oxidative stress. Nephrol Dial Transplant.

[156] Arany Z, Lebrasseur N, Morris C, Smith E, Yang W, Ma Y, Chin S, Spiegelman B (2007). The transcriptional coactivator PGC-1beta drives the formation of oxidative type IIX fibers in skeletal muscle. Cell Metab.

[157] Claro L, Moreno-Amaral A, Gadotti A, Dolenga C, Nakao L, Azevedo M, de Noronha L, Olandoski M, de Moraes T, Stinghen A, Pécoits-Filho R (2018). The Impact of uremic toxicity induced inflammatory response on the cardiovascular burden in chronic kidney disease. Toxins.

[158] Enoki Y, Watanabe H, Arake R, Fujimura R, Ishiodori K, Imafuku T, Nishida K, Sugimoto R, Nagao S, Miyamura S, Ishima Y, Tanaka M, Matsushita K, Komaba H, Fukagawa M, Otagiri M, Maruyama T (2017). Potential therapeutic interventions for chronic kidney disease-associated sarcopenia via indoxyl sulfate-induced mitochondrial dysfunction. J Cachexia Sarcopenia Muscle.

[159] Barazzoni R, Zhu X, Deboer M, Datta R, Culler M, Zanetti M, Guarnieri G, Marks D (2010). Combined effects of ghrelin and higher food intake enhance skeletal muscle mitochondrial oxidative capacity and AKT phosphorylation in rats with chronic kidney disease. Kidney Int.

[160] Tamaki M, Miyashita K, Hagiwara A, Wakino S, Inoue H, Fujii K, Fujii C, Endo S, Uto A, Mitsuishi M, Sato M, Doi T, Itoh H (2017). Ghrelin treatment improves physical decline in sarcopenia model mice through muscular enhancement and mitochondrial activation. Endocr J.

[161] Rodriguez Ayala E, Pecoits-Filho R, Heimbürger O, Lindholm B, Nordfors L, Stenvinkel P (2004). Associations between plasma ghrelin levels and body composition in end-stage renal disease: a longitudinal study. Nephrol Dial Transplant.

[162] Wynne K, Giannitsopoulou K, Small C, Patterson M, Frost G, Ghatei M, Brown E, Bloom S, Choi P (2005). Subcutaneous ghrelin enhances acute food intake in malnourished patients who receive maintenance peritoneal dialysis: a randomized, placebo-controlled trial. J Am Soc Nephrol.

[163] Kunkel S, Elmore C, Bongers K, Ebert S, Fox D, Dyle M, Bullard S, Adams C (2012). Ursolic acid increases skeletal muscle and brown fat and decreases diet-induced obesity glucose intolerance and fatty liver disease. PLoS ONE.

[164] Kunkel S, Suneja M, Ebert S, Bongers K, Fox D, Malmberg S, Alipour F, Shields R, Adams C (2011). mRNA expression signatures of human skeletal muscle atrophy identify a natural compound that increases muscle mass. Cell Metab.

[165] Yu R, Chen J, Xu J, Cao J, Wang Y, Thomas S, Hu Z (2017). Suppression of muscle wasting by the plant-derived compound ursolic acid in a model of chronic kidney disease. J Cachexia Sarcopenia Muscle.

[166] El-Kateb S, Davenport A (2016). Changes in intracellular water following hemodialysis treatment lead to changes in estimates of lean tissue using bioimpedance spectroscopy. Nutr Clin Pract.

[167] Panorchan K, Nongnuch A, El-Kateb S, Goodlad C, Davenport A (2015). Changes in muscle and fat mass with haemodialysis detected by multi-frequency bioelectrical impedance analysis. Eur J Clin Nutr.

[168] Kang S, Cho K, Park J, Yoon K, Do J (2014). Body composition measurements using bioimpedance analysis in peritoneal dialysis patients are affected by the presence of dialysate. Nephrology.

[169] Pinto A, Ramos C, Meireles M, Kamimura M, Cuppari L (2015). Impact of hemodialysis session on handgrip strength. J Bras Nefrol.

[170] Guralnik J, Ferrucci L, Pieper C, Leveille S, Markides K, Ostir G, Studenski S, Berkman L, Wallace R (2000). Lower extremity function and subsequent disability: consistency across studies predictive models, and value of gait speed alone compared with the short physical performance battery. J Gerontol A Biol Sci Med Sci.

[171] Ishikawa S, Naito S, Iimori S, Takahashi D, Zeniya M, Sato H, Nomura N, Sohara E, Okado T, Uchida S, Rai T (2018). Loop diuretics are associated with greater risk of sarcopenia in patients with non-dialysis-dependent chronic kidney disease. PLoS ONE.

[172] Ishii S, Tanaka T, Shibasaki K, Ouchi Y, Kikutani T, Higashiguchi T, Obuchi S, Ishikawa-Takata K, Hirano H, Kawai H, Tsuji T, Iijima K (2014). Development of a simple screening test for sarcopenia in older adults. Geriatr Gerontol Int.

[173] Vettoretti S, Caldiroli L, Armelloni S, Ferrari C, Cesari M, Messa P (2019). Sarcopenia is associated with malnutrition but not with systemic inflammation in older persons with advanced CKD. Nutrients.

[174] Kritmetapak K, Peerapornratana S, Srisawat N, Somlaw N, Lakananurak N, Dissayabutra T, Phonork C, Leelahavanichkul A, Tiranathanagul K, Susantithapong P, Loaveeravat P, Suwachittanont N, Wirotwan TO, Praditpornsilpa K, Tungsanga K, Eiam-Ong S, Kittiskulnam P (2016). The impact of macro-and micronutrients on predicting outcomes of critically ill patients requiring continuous renal replacement therapy. PLoS ONE.

[175] Fried LP, Tangen CM, Walston J, Newman AB, Hirsch C, Gottdiener J, Seeman T, Tracy R, Kop WJ, Burke G, McBurnie MA (2001). Frailty in older adults: evidence for a phenotype. J Gerontol A Biol Sci Med Sci.

[176] Kamijo Y, Kanda E, Ishibashi Y, Yoshida M (2018). Sarcopenia and frailty in PD: impact on mortality malnutrition and inflammation. Perit Dial Int.

[177] Abro A, Delicata L, Vongsanim S, Davenport A (2018). Differences in the prevalence of sarcopenia in peritoneal dialysis patients using hand grip strength and appendicular lean mass: depends upon guideline definitions. Eur J Clin Nutr.

[178] da Silva M, Vogt B, Reis N, Caramori J (2019). Update of the European consensus on sarcopenia: what has changed in diagnosis and prevalence in peritoneal dialysis?. Eur J Clin Nutr.

[179] Yanishi M, Kinoshita H, Tsukaguchi H, Kimura Y, Koito Y, Sugi M, Matsuda T (2018). Factors related to osteosarcopenia in kidney transplant recipients. Transplant Proc.

[180] Macdonald J, Marcora S, Jibani M, Roberts G, Kumwenda M, Glover R, Barron J, Lemmey A (2006). Bioelectrical impedance can be used to predict muscle mass and hence improve estimation of glomerular filtration rate in non-diabetic patients with chronic kidney disease. Nephrol Dial Transplant.

[181] Wilkinson T, Nixon D, Richler-Potts D, Neale J, Song Y, Smith A (2019). Identification of the most clinically useful skeletal muscle mass indices pertinent to sarcopenia and physical performance in chronic kidney disease. Nephrology (Carlton).

